# Genome-wide association study of traits in sacred lotus uncovers MITE-associated variants underlying stamen petaloid and petal number variations

**DOI:** 10.3389/fpls.2022.973347

**Published:** 2022-09-23

**Authors:** Zhiyan Gao, Yuting Liang, Yuhan Wang, Yingjie Xiao, Jinming Chen, Xingyu Yang, Tao Shi

**Affiliations:** ^1^Key Laboratory of Aquatic Botany and Watershed Ecology, Wuhan Botanical Garden, Chinese Academy of Sciences, Wuhan, China; ^2^Center of Conservation Biology, Core Botanical Gardens, Chinese Academy of Sciences, Wuhan, China; ^3^College of Life Sciences, University of Chinese Academy of Sciences, Beijing, China; ^4^Wuhan Institute of Landscape Architecture, Wuhan, China; ^5^Wuhan Institute of Design and Sciences, Wuhan, China; ^6^National Key Laboratory of Crop Genetic Improvement, Huazhong Agricultural University, Wuhan, China

**Keywords:** GWAS, MITEs, sacred lotus, stamen petaloid, petal number

## Abstract

Understanding the genetic variants responsible for floral trait diversity is important for the molecular breeding of ornamental flowers. Widely used in water gardening for thousands of years, the sacred lotus exhibits a wide range of diversity in floral organs. Nevertheless, the genetic variations underlying various morphological characteristics in lotus remain largely unclear. Here, we performed a genome-wide association study of sacred lotus for 12 well-recorded ornamental traits. Given a moderate linkage disequilibrium level of 32.9 kb, we successfully identified 149 candidate genes responsible for seven flower traits and plant size variations, including many pleiotropic genes affecting multiple floral-organ-related traits, such as *NnKUP2*. Notably, we found a 2.75-kb presence-and-absence genomic fragment significantly associated with stamen petaloid and petal number variations, which was further confirmed by re-examining another independent population dataset with petal number records. Intriguingly, this fragment carries MITE transposons bound by siRNAs and is related to the expression differentiation of a nearby candidate gene between few-petalled and double-petalled lotuses. Overall, these genetic variations and candidate genes responsible for diverse lotus traits revealed by our GWAS highlight the role of transposon variations, particularly MITEs, in shaping floral trait diversity.

## Introduction

Understanding the genetic and molecular basis of phenotypic diversity among individuals within species is a long-standing problem in evolutionary developmental biology and population genetics (Huang and Han, 2014; Payne and Wagner, 2019). Previously, it was widely accepted that genetic variation, including single nucleotide variants and structural variants, in protein-coding regions directly alters protein structures and functions to control developmental complexity and phenotypic variation (Syvanen, 2001; Shastry, 2009). Currently, growing empirical evidence highlights the predominant role of variations in non-coding regulatory regions in affecting morphological variations in animals and plants (Glazier et al., 2002; Carroll, 2005; Hoekstra and Coyne, 2007; Romero et al., 2012; Signor and Nuzhdin, 2018). To detect significant associations between genotypes and phenotypic variance in diverse populations, a genome-wide association study (GWAS) based on linkage disequilibrium (LD) has become a powerful approach to detect many natural allelic variations simultaneously in a single study (Hayes, 2013). GWASs of different crops have discovered critical molecular markers containing single nucleotide polymorphism (SNP) loci and candidate gene regions associated with structural and physiological traits in maize (Tian et al., 2011), tomato (Lin et al., 2014), soybean (Zhang et al., 2015), and rice (Yano et al., 2016). Intriguingly, many recent GWASs of different plant systems have also found that transposon insertions, particularly miniature inverted-repeat transposable elements (MITEs), play an important role in trait diversity by affecting the expression or structural change of nearby genes. In rice, a transposon-insertion-polymorphisms-GWAS revealed that the insertions of four significant MITEs located inside key genes or in their close vicinity were associated with six grain-related phenotypes, including grain width (Castanera et al., 2021). In another GWAS example, a 125 bp MITE insertion in the promoter region of the *TaVSR1-B* gene suppressed the expression of this gene, leading to longer and thinner roots of wheat at the booting stage (Wang et al., 2021). In addition, a GWAS based on presence and absence variations detected a 621 bp MITE that was inserted in the promoter region and enhanced the expression of *FLC,* thus enhancing the vernalization of winter-type oilseed rapes (Song et al., 2020). These pieces of evidence seem to show that transposon variations are one of the driving forces underlying phenotypic diversity in plant adaptation and domestication.

Floral traits, such as flower shapes in *Arabidopsis* (Takeda et al., 2013), flower sizes in *Penstemon* (Parachnowitsch and Kessler, 2010) and flower colors in *Petunia* (Tornielli et al., 2009), are diverse and often desirable for horticulture. Understanding the genetic variations underlying floral morphological differentiation is a crucial task for both plant developmental biology and horticulture research, particularly for ornamental flowers, such as chrysanthemum, rose and sacred lotus. Although parts of the biosynthesis pathways regulating floral development have been verified to be relatively conserved across model plants (Litt and Kramer, 2010; Ruiz-Sola and Rodriguez-Concepcion, 2012; Saito et al., 2013), several studies on non-model plant species are needed to verify and investigate the multitude of functional alleles related to flowering time, floral shapes, pigmentation and petal numbers based on GWAS. For example, an SNP in the *PPR* gene was linked to flowering time leading to early- and late-flowering pearl millets (Diack et al., 2020). In another example, both known and novel genes associated with petal size were identified in rapeseed (Qian et al., 2021). Some flower color variants were also identified by GWAS case studies. In *Prunus mume* and rose, several known candidate genes (*4CL*) were verified, and some new candidate genes (*CDC6*) were found to affect anthocyanin and carotenoid concentrations (Schulz et al., 2016; Zhang et al., 2018). In addition, modeling multiple flower traits with polymorphisms together in one study could improve the prediction accuracy of key genes underlying trait variations when compared with the study of one trait independently, especially for correlated plant development traits. In *Petunia*, there were significant correlations between flower length, flower diameter, flower number and florescence, and multiple SNP loci were colocalized in four intervals on four linkage groups controlling various traits (Cao et al., 2018). Therefore, the study of multiple flower traits in a single GWAS could help to comprehensively reveal the mechanisms underlying the diversity of complex developmental traits.

Lotus (*Nelumbo nucifera* Gaertn or sacred lotus) is a famous and traditional aquatic flower with significant cultural and religious symbolization in ancient civilizations, such as China and India. It exhibits diverse and attractive floral traits with thousands of years of gardening (Wang, 2005). Lotus can be classified into three main groups according to their primary utilizations, namely rhizome lotus, seed lotus, and flower lotus (Wang, 2005; Lin et al., 2019). Flower lotus has been widely cultivated in Asia because of its diverse flower coloration and complex floral morphologies (Guo, 2008). The domestication history of flower lotus from few-petalled red flowers to double-petalled, duplicate-petalled, and all-double-petalled flowers can be traced back to China’s Jin dynasty. With the deepening of cultural communication and the development of cultivation techniques in lotus, the all-double-petalled lotus was cultivated in the Northern and Southern Dynasties of China (Wang, 2005; Guo, 2008; Liu et al., 2020). A broad spectrum of flower colors and patterns from white to red exists in lotus, which is associated with the amount and types of anthocyanin affected by various epigenetic regulators and the expression of structural genes (such as *CHI*, *UFGT*, *ANS*, *FLS*, *OMTs*, and *GST*; Deng et al., 2015; Sun et al., 2016; Wang et al., 2016; Zhu et al., 2019) and transcription factors (such as *MYB5* and *TT8*; Sun et al., 2016; Deng et al., 2021). Generally, the types of lotus flowers vary from few-petalled flowers to various double-petalled flowers because flowers with stamen petaloid and carpel petaloid fail to form normal floral patterns. These processes have been predicted to be influenced by several hormone-related candidate genes and transcription factors (TFs; Lin et al., 2018), and epigenetic regulation (methylation and miR172 family; Lin et al., 2019; Zhang Y. et al., 2020). The chromosomal-level genome assembly of lotus cv. ‘China Antique’ (Ming et al., 2013; Shi et al., 2020) provides opportunities for the population, expression and genomic studies of its natural variations, such as in rhizome enlargement (Yang et al., 2015; Gao et al., 2021; Huang et al., 2021; Li et al., 2021a), seed development (Li et al., 2018) and flower development (Lin et al., 2019). Using a reference genome and transcriptomic analysis, some lotus genes were also predicted to control the flowering time of lotus by participating in the photoperiod, vernalization and gibberellic acid pathways (Yang et al., 2014; Zhang et al., 2021). Although previous transcriptomic or methylation genomic studies revealed the molecular mechanisms underlying a single trait for flower development, no systematic study has determined the genetic architecture of multiple correlated traits in a population using GWAS in lotus. In this study, to better uncover the genetic variants and molecular mechanisms underlying the various traits in the cultivated flower lotus, we comprehensively performed GWAS with the generalized linear model (GLM). After further investigation of the variants in gene structure, the expression patterns of candidate genes, and the transposable elements (TEs) and small RNAs associated with floral diversity and plant size, we provide insights into the genetic basis underlying crucial ornamental trait variations of this traditional flower.

## Materials and methods

### Plant materials

A total of 88 cultivated lotus accessions with abundant diversity in phenotypic traits were grown in pots (50 cm × 50 cm) under the same conditions at the Wuhan Institute of Landscape Architecture (114°52′ E, 30°50’ N), Wuhan, China. Twelve crucial phenotypic traits, including flowering time, population florescence, flower density, petal density, flower type, stamen petaloid, flower color, flower shape, flower diameter, maximum petal length, maximum petal width, and plant size, were investigated and confirmed by at least 3 years of observations during long-term cultivation, and used for GWAS (Supplementary Table S1; Wang, 2005; Wang and Zhang, 2005; Guo, 2008; Zhang and Wang, 2011). The rationale of choosing these 88 lotus cultivars in our GWAS is because (1) they are registered cultivars while registration requires at least three consecutive years of observation on stable traits, (2) they have been cultivated and validated for trait stability for at least 3 years and only cultivars with traits consistent with registration records were remained for conservation, and (3) their key phenotypes we focused on this study have been already recorded in ‘*Lotus Flower Cultivars In China*’ (Wang, 2005), ‘*Colored Illustration of Lotus Cultivars in China*’ (Wang and Zhang, 2005), ‘*Cultivation Of Lotus (Nelumbo Nucifera Gaertn. Ssp. Nucifera) And Its Utilization In China*’ (Guo, 2008), and ‘*New Lotus Flower Cultivars in China I*’ (Zhang and Wang, 2011). All trait data in GWAS were based on the records of the books and our validations, and deposited in *Nelumbo Genome Database* (Shi et al., 2020; Li et al., 2021b).^1^

### SNP calling, validation, and annotation

To explore genetic variations in this lotus germplasm, the resequencing data for the abovementioned 88 accessions were previously sequenced in an Illumina platform with more than 10× coverage depth for each sample and deposited in *Nelumbo Genome Database* (Shi et al., 2020; Li et al., 2021b). Resequencing reads were mapped to the reference lotus genome ‘China Antique’ from our *Nelumbo Genome Database* (Shi et al., 2020; Li et al., 2021b) with BWA (v0.7.12) using the default parameters (Li, 2013). Nearly 99.14% of clean reads were mapped to the reference genome, with an average sequencing depth of 14.93-fold (Supplementary Table S2). These mapping outputs of the 88 accessions in SAM format were then converted into the BAM format and coordinate sorted using SAMtools (version 0.1.19)^2^ and Picard (version 2.0.1).^3^ Subsequently, SNP calling was carried out *via* the Genome Analysis Toolkit (GATK 4; McKenna et al., 2010) using a standard pipeline^4^ to obtain single-sample gvcf files. High-quality SNPs in the joint genotyping data were retained for subsequent analyses by restricting the missing rate (−max-missing 1), minor allele frequency (−MAF 0.05), number of alleles (−min-alleles 2, −max-alleles 2) and read depth (−minDP 3, −-maxDP 100, −min-meanDP 3) for each SNP locus. These high-quality SNPs were annotated using SnpEff^5^ based on the reference genome.

### Population genetic analyses

The high-quality SNPs with an *r*^2^ > 0.5 (pairwise coefficient) within 100 kb windows were pruned to avoid the effects of excessive LD between adjacent SNP loci using Plink 1.9 (Purcell et al., 2007). PopLDdecay v3.31 with MaxDist set at 1,000 was used to calculate and plot the LD decay of each group (Zhang et al., 2019). The population genetic structure was determined by ADMIXTURE with cross-validation (CV) of the hypothetical number of populations (*K*) ranging from 1 to 10 based on a pruned set of SNPs (Alexander et al., 2009). The population structure was also assessed by principal component analysis (PCA) with Genome-Wide Complex Trait (GCTA) using pruned SNPs (Yang et al., 2011). To further provide insight into the population’s genetic structure, the phylogenetic relationships among the 88 cultivated lotus samples were analyzed using SNPhylo software (Lee et al., 2014).

### Genome-wide association and candidate gene analyses

A GWAS was performed with 455,292 high-quality non-missing SNPs and 12 traits from 88 accessions in TASSEL (v5.2.44)^6^ based on a generalized linear model (GLM). The GWAS results were visualized in the form of Manhattan and QQ plots using R (v3.31). The significantly associated SNP loci were estimated based on significance level (*, α = 0.05, −log_10_ *p* = 6.96, *p* value <1.106378e-07) and corrected by Benjamini–Hochberg correction to reduce false-positive associations. According to the LD blocks of the association population, genes within ±50 kb of the significantly associated SNP loci were identified. Homologous genes in *Nelumbo* were annotated with the highest e-value based on the TAIR10 dataset^7^ using BlastP v.2.2.31. The functions of homologous and nonhomologous genes were found in the *Arabidopsis* and *Nelumbo* databases. The expression profiles of candidate genes were collected from the *Nelumbo Genome Database*. The phylogenetic relationships of these orthologous protein sequences among lotus, *Arabidopsis* and *Oryza sativa* were constructed by the neighbor-joining (NJ) approach with 500 bootstrap replicates using MEGA v.7.0 after alignment with MUSCLE with default settings. The gene networks were visualized using Cytoscape v3.5.1 based on a weighted gene coexpression network (WGCNA) from the *Nelumbo Genome Database* to depict the relationships between genes. In addition, known lncRNAs, small RNAs, and TEs in previous studies were also identified by Bedtools (Shi et al., 2017, 2020).

To verify the association between the stamen petaloid trait and genotype variants, the sequencing coverages and genotypes of the deletion region on chromosome 5 across 88 lotus accessions examined in our study and 136 selected lotus accessions from Liu et al. (2020; with an Illumina sequencing coverage depth > 5) were calculated by Bedtools and Delly, respectively (Quinlan and Hall, 2010; Rausch et al., 2012). In addition, transcriptome datasets from petals of double-petalled lotus (‘Fenhonglingxiao’; Lin et al., 2018) and few-petalled lotus (‘China Antique’) from the *Nelumbo Genome Database* were used to find expression differences between them for candidate genes close to the deletion region with StringTie (Pertea et al., 2016) and DESeq2 (|log2FC| > 1 and Padj <0.05; Love et al., 2014).

## Results

### Genome-wide SNP detection and population parameters of flower lotus

**Figure 1:**
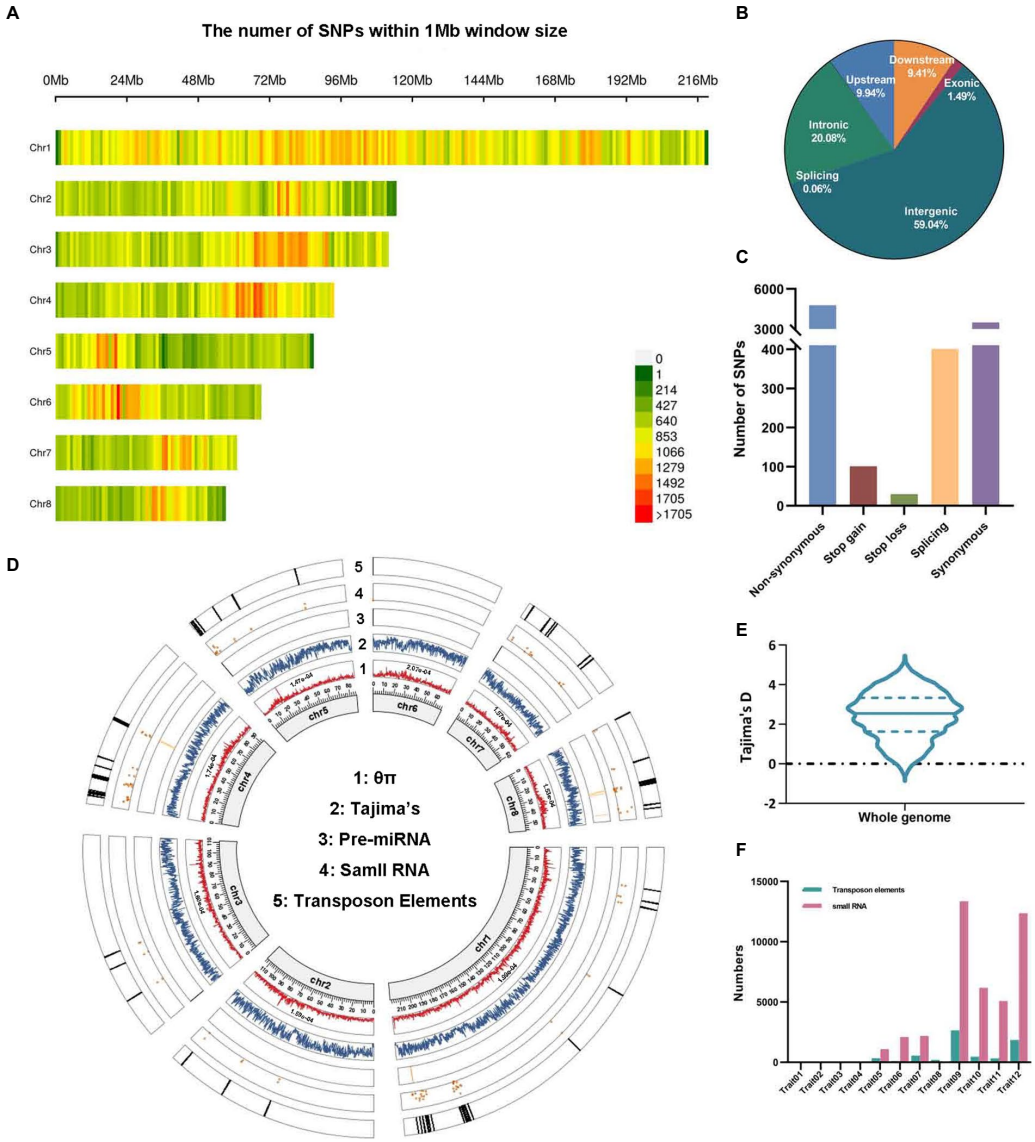
The analysis of 455,292 high-quality SNPs in 88 lotus accessions. **(A)** SNP density across eight chromosomes. **(B,C)** The genomic distributions **(B)** and functional annotations **(C)** of SNPs in 88 lotus accessions. **(D)** Genetic and genomic features of SNPs on each chromosome are shown in a Circos plot. 1: Mean nucleotide diversity (θπ). 2: Tajima’s D. 3–5: The distribution patterns of known pre-miRNA, small RNA (sRNA) and transposon elements (TEs) significantly linked to SNPs from other studies. **(E)** Tajima’s D values across the whole genome. **(F)** The proportion of sRNA and TEs associated with 12 traits in 88 lotus accessions.

To ensure the accuracy of GWAS, it is important to select an effective population with sufficient genotypic and phenotypic diversity. A final set of 455,292 high-quality SNPs were detected based on the resequencing of 88 cultivated lotus individuals with diverse recorded traits, which were almost uniformly distributed across the eight lotus chromosomes (Figure 1A; Supplementary Tables S1, S2). Among these SNPs, 230,995 (40.96%) were located within genes, and the remainder were located in intergenic regions (59.04%; Figure 1B). In the coding regions, more than 5,300 SNPs (1.2% of the total) were predicted as nonsynonymous (4,771), stop-gain (101), stop-loss (30), and splicing (401) variants that have a potential effect on amino acid changes, elongated transcripts or premature stop codons (Figure 1C). This percentage is lower than those for watermelon (5.6%; Guo et al., 2019) and rice (10.1%; Yano et al., 2016) but close to those for maize (1.9% Jiao et al., 2012) and cotton (1.3%; Ma et al., 2018). Furthermore, 14,435 genes were found to have SNPs in their coding sequences (CDSs). In contrast, 20,051 genes without SNP variations were found, suggesting that their CDS was highly conserved. The above results demonstrate that the SNP dataset in our study can provide abundant genetic markers for resolving the genetic mechanisms underlying various traits through GWAS.

We further assessed genome-wide nucleotide diversity (θπ), revealing relatively higher average nucleotide diversity (θπ) on chromosome 6 (θπ = 2.07e-04) and chromosome 1 (θπ = 1.99e-04) than on the other chromosomes across the 88 cultivated lotuses (Figure 1D). In addition, Tajima’s D was greater than zero, suggesting overall balancing selection or sudden contraction of the lotus population size, which likely preserved multiple beneficial alleles (Figures 1D,E).

Population structure and PCA were performed to evaluate genetic differentiation among samples based on 155,564 SNPs after removing redundant SNPs in LD blocks. These 88 accessions were predicted to be derived from a single ancestral population, as the lowest cross-validation error was found when *K* = 1 (Supplementary Figure S1A). In addition, our PCA plot showed a continuous distribution of lotus individuals without distinct clusters, which was concordant with our population structure analysis (Supplementary Figure S1B). Besides, the average proportion of heterozygosity for the whole lotus population was 0.224. 74 out of 88 lotus individuals showed observed heterozygosity ranging from 0.20 to 0.30, indicating that our population had high genetic diversity owing to extensive hybridization during breeding (Supplementary Figure S1C). The phylogenetic tree indicated that the 88 lotus accessions were indeed from one population because of extremely low bootstrap values (lower than 27) in early-branching nodes, which cannot confidently divide individuals into different populations (Supplementary Figure S2). Approximately 48.86% of the bootstrap values in this phylogenetic tree were less than 80, indicating that the phylogenetic tree had low statistical support (Supplementary Figure S1D). Therefore, the overall cultivated flower lotuses did not show strong population structure or population divergence, indicating that population structure might not affect GWAS analysis. Importantly, half of the pairwise correlation coefficient (*r*^2^) maximum value (*r*^2^ = 0.42) at 32.9 kb was taken as the critical value for all lotus accessions after calculating the extent of LD (Supplementary Figure S1E), which was lower than that for the flower lotus groups (58 kb) from a previous lotus study (Liu et al., 2020). This result suggested that our lotus population might have more extensive recombination through hybridization and crossing, leading to shorter linkage blocks. All the above genetic indices supported that the 88 cultivated lotuses were suitable for a GWAS because of their moderate LD decay rate, low population structure and high phenotypic diversity.

### Genotypic characterization and SNP loci associated with traits

**Figure 2:**
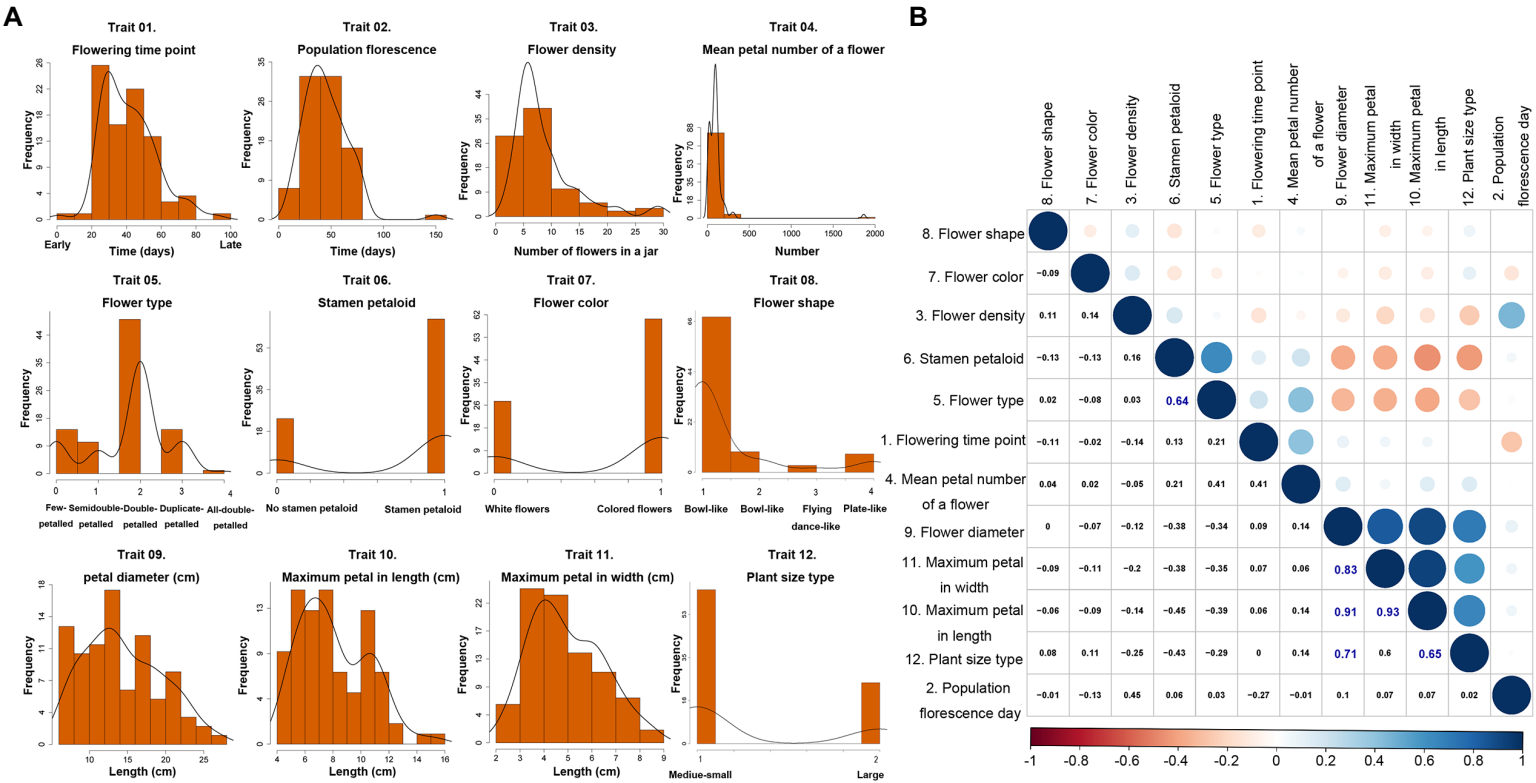
Diversity and relationships of 12 traits in 88 lotus accessions. **(A)** Frequency distribution of 12 traits in 88 accessions. **(B)** The pairwise *Pearson* correlations among 12 traits in 88 lotus accessions.

Here, we investigated the 12 key important traits of 88 lotus accessions (Figure 2; Supplementary Table S1). The seven quantitative traits, including flower time, flower density and flower type, showed relatively normal distributions. However, the remaining quality traits, like stamen development and flower color, varied markedly among these samples (Figure 2A). The Pearson correlation analysis revealed that there were moderate to strong positive correlations between stamen petaloid and flower type (*r* = 0.64, value of *p* = 2.682e-05), between maximum petal length and plant size (*r* = 0.65, value of *p* = 2.597e-05), between flower diameter and plant size (*r* = 0.71, value of *p* = 8.978e-06), between maximum petal width and flower diameter (*r* = 0.83, value of *p* = 3.794e-09), between maximum petal length and flower diameter (*r* = 0.91, *p* value = 4.203e-11), and between maximum petal width and maximum petal length (*r* = 0.93, value of *p* = 2.422e-11), which suggested that some traits are strongly linked during lotus flower or plant development (Figure 2B).

**Figure 3:**
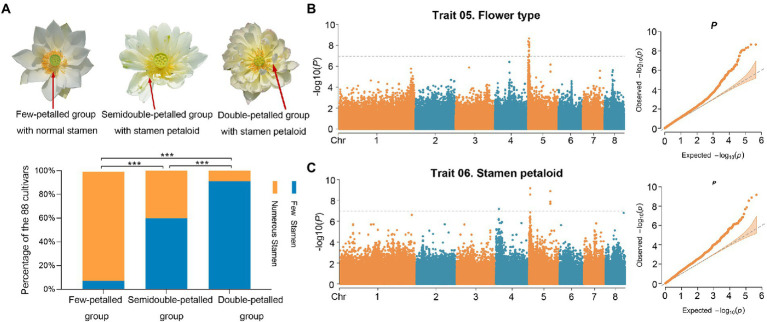
GWAS of flower type and stamen petaloid. **(A)** The relationship between stamen numbers and flower types with petal numbers in different groups (***, value of *p* < 0.0001). The three images displayed flowers in lotus with different flower types and stamen petaloid numbers. Red arrows point to the stamens and stamen petaloid. **(B,C)** Manhattan (left) and QQ (right) plots of GWAS results for flower type **(B)** and stamen petaloid **(C)** using a generalized linear model (*GLM*). The gray dotted lines and gray solid lines represent significance levels [***, −log10(*P*) = 6.9, value of *p* = 1.106378e-07].

Furthermore, using 455,292 high-quality SNPs, a GWAS analysis for these 12 traits was carried out using a generalized linear model (GLM) with controlling for population structure (*K* = 1). QQ-plots that displayed the distribution of calculated *p* value were similar to the theoretical *p* value in the expected diagonal, indicating the accuracy of these GWAS results (Figure 3; Supplementary Figure S3). Intriguingly, 10 out of the 12 traits had a total of 438 significantly associated SNPs given the significance threshold after excluding the repeated SNP loci based on GLM (value of *p* <1.106378e-07; Figure 3; Supplementary Figure S3, Supplementary Table S3). Of these 438 SNPs, the number of SNPs in non-coding regions (72.15%) was two times higher than that in coding regions (27.85%; Supplementary Table S3). Flower diameter had the highest number of associated SNPs (46.58% of the total significant SNPs), followed by plant size (21.46%) and maximum petal length (11.87%; Supplementary Figure S3, Supplementary Table S3). Moreover, the numbers of LD blocks of different lengths were summarized across the whole genome, and we identified that the length of most LD blocks was 50–100 kb (Supplementary Figure S1F). Thus, we examined ±50 kb regions surrounding each of the 70 representative SNPs; these regions contained 149 candidate genes associated with phenotypes (Table 1; Supplementary Table S4). In the non-coding regions surrounding these candidate SNPs associated with the phenotypes, 28,764 associated non-coding RNAs (ncRNAs) and 6,317 TEs were found, which were frequently distributed on chromosome 1, encompassing 27.84% of the ncRNAs and 30.82% of the TEs (Figures 1D,F; Supplementary Tables S5, S6).

**Table 1:** The number of candidate coding genes significantly associated with flower types and stamen petaloid in 88 lotus accessions.

**Trait**	**SNP Marker**	***P* alue**	**Lotus GeneID**	***Arabidopsis*_ID (Name)**	**Function**
**Trait 05. Flower type**	chr5-897,119	4.1826E-09	*Nn5g26732*	*AT2G38670* (*PECT1*)	Phosphatidylethanolamine biosynthetic process.
	chr5-3,675,283	4.9692E-09	*Nn5g26876*	*#N/A*	#N/A
			*Nn5g26877*	*#N/A*	Transcription repressor *OFP1*-like, DNA-binding.
			*Nn5g26878*	*#N/A*	#N/A
			*Nn5g26879*	*AT2G30395* (*OFP17*)	Member of the plant-specific ovate protein family.
			*Nn5g26880*	*AT2G40540* (*KUP2*)	Putative potassium transporter *AtKT2p* (*AtKT2*) mRNA, potassium ion transport.
	chr5-1,819,278	8.4980E-09	*Nn5g26787*	*AT5G01960*	Ubiquitin protein ligase activity.
	chr5-2,290,307	3.5133E-08	*Nn5g26803*	*AT5G58290* (*RPT3*)	Positive regulation of RNA polymerase II transcription preinitiation complex assembly, protein catabolic process, ubiquitin-dependent protein catabolic process.
**Trait 06. Stamen petaloid**	chr5-3,675,283	6.9103E-10	*Nn5g26876*	*#N/A*	#N/A
			*Nn5g26877*	*#N/A*	Transcription repressor *OFP1*-like, DNA-binding.
			*Nn5g26878*	*#N/A*	#N/A
			*Nn5g26879*	*AT2G30395* (*OFP17*)	Member of the plant-specific ovate protein family.
			*Nn5g26880*	*AT2G40540* (*KUP2*)	Putative potassium transporter *AtKT2*p (*AtKT2*) mRNA, Potassium ion transport.
**Trait 06. Stamen petaloid**	chr5-61,775,841	1.3503E-08	*Nn5g29478*	#N/A	Negative regulation of organ growth, ovule morphogenesis, seed morphogenesis, ubiquitin-binding, zinc ion binding.
			*Nn5g29482*	*AT3G50770* (*CML41*)	Calcium-binding protein.
			*Nn5g29483*	#N/A	#N/A
			*Nn5g29484*	*AT4G34350* (*HDR*)	Dimethylallyl diphosphate biosynthetic process, isopentenyl diphosphate biosynthetic process, methylerythritol 4-phosphate pathway.
			*Nn5g29485*	*AT3G47850*	*MYB* proto-oncogene protein, plant (A).

**Figure 4:**
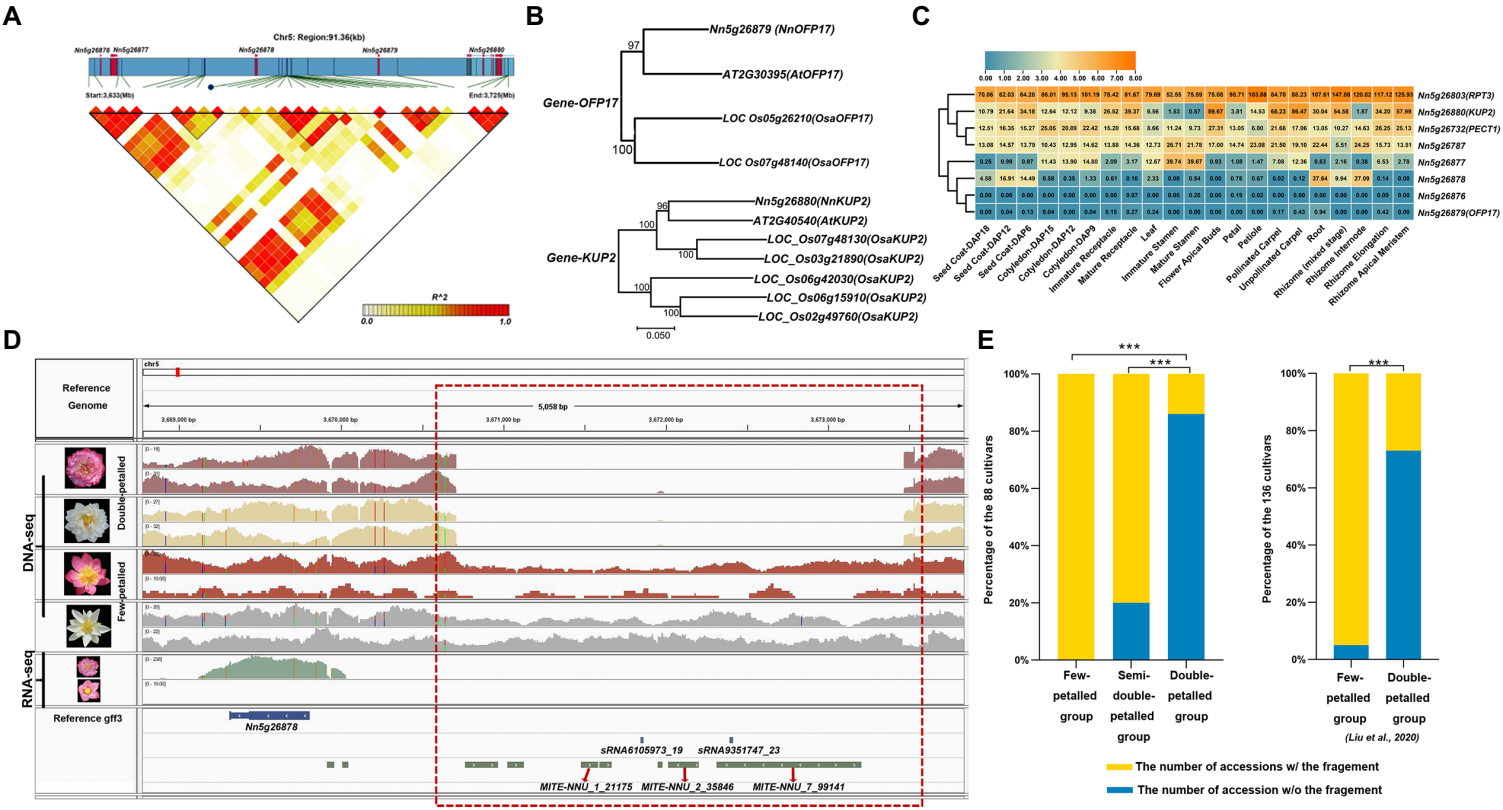
The candidate genes associated with flower types and stamen petaloid. **(A)** The regions of low-to-high LD, measured by the *r*^2^ statistic, are represented by a color gradient. **(B)** The two phylogenetic trees of candidate orthologous genes (*OFP17* and *KUP2*) associated with flower types between lotus (*Nn*), *Arabidopsis* (*At*) *and Oryza sativa* (*Osa*). And the neighbor-joining (NJ) trees with 500 bootstrap replicates was constructed by MEGA 7.0. Numbers at nodes indicate bootstrap values. **(C)** Heatmap and hierarchical clustering of the candidate genes showing the expression pattern in different tissues. **(D)** Integrative genomics viewer displaying the long-gap variant (chr5: 3,670,710-3,673,465, area within the red box) near a candidate gene with differential expression between few-petalled and double-petalled lotus. **(E)** The proportions of lotus accessions with genomic fragments that are completely deleted (blue) or present (yellow) in the region (3,670,710–3,673,465 on chromosome 5) out of the 88 lotus individuals examined in our study (left panel) and the 136 lotus accessions with high-depth resequencing datasets from Liu et al. (2020) (right panel) based on Illumina read coverage (***, value of *p* < 0.001).

To gain insight into the functions of these trait-related candidate genes, we investigated the functional annotation of homologous genes in *Arabidopsis* and gene expression from various lotus tissues in the *Nelumbo Genome Database*. Among these genes, 25, 32 and 17 genes with known functions were distributed on chromosome 1, chromosome 4 and chromosome 7, respectively (Supplementary Figure S4). Further hierarchical clustering analysis of the gene expression profiles of various tissues for candidate genes can distinguish genes into several groups with modest yet consistent expression patterns, suggesting that such genes might share similar functions (Figure 4; Supplementary Figure S5; Eisen et al., 1998).

### Candidate genes for flower types and stamen petaloid

Stamen petaloid and flower type, which mainly characterize petal layers and numbers, are two significantly correlated traits (Figure 2B). Notably, in these 88 lotus accessions, the number of stamens was inversely related to the number of petals through pairwise comparisons among the few-petalled lotus group, semidouble-petalled group and double-petalled group (χ^2^ test of independence, value of *p* <0.001; Figure 3A). In the few-petalled lotus group, 92.86% had a few stamens, while the remaining flowers (7.14%) had numerous stamens; in the semidouble-petalled group, 60% of the accessions had a few stamens, while 40% had numerous stamens; and in the remaining groups with numerous petals, up to 86.49% exhibited few or extremely few stamens due to the petalled stamens. Our GWAS model uncovered five meaningful SNPs associated with 13 genes in three LD blocks for the two traits (Figures 3B,C; Table 1; Supplementary Table S4). The SNP locus (chr5_3,675,283) within the strong peak signal on chromosome 5 was associated with both stamen petaloid and flower type (Table 1; Figure 4A). To determine the expression differences in these genes between double-petalled and few-petalled flower groups, transcriptomes of lotus ‘China Antique’ (few-petalled) versus cultivated ‘Xiaoxia’ (double-petalled) at the formation period of flower buds and flower blooming stage were compared. Moreover, the differences in gene expression among petal, normal stamen, and stamen petaloid tissues in the same double-petalled individual, ‘Fenhonglingxiao’, were also compared to identify tissue-specific genes.

Among the five genes in the candidate region near this SNP locus (chr5_3,675,283), *OFP17* (*Nn5g26879*) and *KUP2* (*Nn5g26880*) have defined orthologs in *Arabidopsis*, while *Nn5g26876, Nn5g26877* and *Nn5g26878* do not have any defined orthologs (Figure 4B; Table 1). *KUP2,* with a function in shoot cell expansion, was preferentially expressed in the flower apical buds of the few-petalled group (Figures 4C; Supplementary Figure S5A; Elumalai et al., 2002). However, in the double-petalled group, it was expressed the highest in stamen petaloid (Supplementary Figure S6A). The expression of *KUP2* in the apical buds and petals in the few-petalled lotus group was twofold that in the double-petalled lotus group, which might be considered that high expression of *KUP2* is likely required for proper petal development (Supplementary Figure S6B). The function of *Nn5g26877* was annotated as the transcriptional repressor *OFP1*-like, which was highly expressed in immature and mature stamen tissues in the few-petalled group but barely expressed in stamen tissue in the double-petalled group, which suggested that its expression might promote the development of normal stamens in the few-petalled group, whereas the absence of expression in the double-petalled group might play a negative role in normal stamen development (Figure 4C; Supplementary Figures S5A, S6A). In line with the above conclusion, from apical buds to petals, the relative expression of this gene in the double-petalled group was threefold higher than that in the few-petalled group (Supplementary Figure S6B). These results implied that the expression of *KUP2* might contribute to stamen petaloid, while the expression of *Nn5g26877* might be involved in the development of normal stamens.

Among other associated genes for flower type and stamen petaloid, *PECT1* (*Nn5g26732*), which can modulate the interaction between phosphatidylcholine and *flowering locus T*, is expressed highest in flower apical buds in both the few-petalled and double-petalled groups (Figure 4C; Supplementary Figures S5; Susila et al., 2021). *Nn5g29478* was predicted to negatively regulate the formation of organ growth. It is expressed at the highest level in petals in both the few-petalled and double-petalled groups (Supplementary Figure S5A; Table 1). In the same few-petalled individual, *CML41* (*Nn5g29482*) expressed higher in stamen than petal tissues (Figure 4C; Supplementary Figure S4A). In comparison among stamens, stamen petaloid and petals within the same double-petalled individual, *CML41* expression was also the highest in normal stamens (Supplementary Figure S6A). However, when comparing the apical flower buds and fully opened flowers between few-petalled and double-petalled individuals, *CML41* always had higher expression in double-petalled accessions (Supplementary Figure S6B). Thus, the relatively higher expression of *CML41*, as a marker stamen-associated gene, in petals of double-petalled compared with few-petalled accessions might suggest that petals from double-petalled flowers might originate from a mutated stamen.

Besides, we found a total of 2,100 small RNAs and 452 TEs associated with flower type and stamen petaloid, which might play roles in gene regulation (Figure 1F; Supplementary Tables S5, S6). Intriguingly, we found that stamen petaloid in double-petalled flowers differed from that in few-petalled flowers due to the deletion of a 2,755 bp DNA fragment (3,670,710–3.673,465) on chromosome 5 upstream of the *Nn5g26878* gene, which we named the lotus *Petaloid-Related Formation* (*PRF*; Figure 4D). Within this region, there are seven TEs (three MITEs) and two small RNAs (19 nt- and 23 nt-sRNAs). In our study, all 14 few-petalled lotus accessions, accounting for 15.91% of the 88 lotus accessions, retained the complete fragment in this region (χ^2^ test of independence, value of *p* <0.001). Approximately 85.94% of the accessions in the double-petalled group completely lost this key genomic segment, which was significantly higher than that in both the few-petalled (0%) and semidouble-petalled (20.00%) lotus groups (χ^2^ test of independence, value of *p* <0.001; Figure 4E). In addition, to further confirm whether the deletion occurring in this region in other lotus accessions is associated with petaloid stamens, we selected 136 accessions with sufficient read depth and a record of petal characteristics and uncovered a deletion in this region by BEDtools (Liu et al., 2020). In line with our result, 58.82% (10 of 17 double-petalled lotuses) displayed complete deletions in the same region on chromosome 5, whereas 94.12% (112 of 119 few-petalled lotuses) showed the complete presence of this genomic fragment (χ^2^ test of independence, value of *p* <0.001; Figure 4E). In parallel, we genotyped this presence and absence variant with Delly. The probability of missing genotypes within double-petalled lotus (95.31%) was significantly higher than that within few-petalled lotus (21.43%) and semidouble-petalled lotus (0.00%) based on our 88 lotus accessions (χ^2^ test of independence, value of *p* <0.001; Supplementary Figure S7A). In the other 136 public accessions, the probability of missing genotypes with double-petalled lotus was 58.82%, whereas that with few-petalled lotus was only 2.52% (χ^2^ test of independence, value of *p* <0.001; Supplementary Figure S7B). Our investigation into the two independent datasets suggested that the absence of this region is likely responsible for double-petalled flowers. Interestingly, the expression of *PRF*, which is most tightly linked to this region, was only found in the petals of double-petalled flowers but not in those of few-petalled flowers (Figure 4D). The relative expression of this gene in the petals of double-petalled flowers was almost 30-fold that in few-petalled flowers, suggesting that the expression of *PRF* can induce the formation of double-petalled flowers (Supplementary Figure S6B). In the same double-petalled cultivated lotus, the *PRF* gene was highly expressed in normally developed stamens (FPKM = 3.82) relative to its expression in the petaloid stamens (FPKM = 1.49) and petals (FPKM = 0.89) of this petaloid cultivar (Supplementary Figure S6A). These results collectively suggested that this *PRF* gene was abundantly expressed in double-petalled flowers and likely contributed to petaloid formation.

### Candidate genes for flower color and size variations

Four lotus genes were found in the candidate regions within LD blocks surrounding three significant SNPs based on GWAS. These genes were homologous to *Arabidopsis NOG1-1*, *TPPG*, *DJ1D* and *RABF2B* and were found to be associated with petal color variation (Supplementary Figures S3E, S5B; Supplementary Table S4). In *Arabidopsis*, these four genes play central roles in the ABA pathway, trehalose biosynthetic process, lactoylglutathione lyase activity, vacuole organization and transportation (Ueda et al., 2004; Ponnu et al., 2011; Kwon et al., 2013; Lee et al., 2017). Notably, the lotus *RABF2B* showed relatively high expression in flower apical buds and petals of the red lotus, ‘China Antique’, which might help to transport anthocyanins to vacuoles (Supplementary Figure S5B). In addition to protein-coding genes with known functions, 2,192 ncRNAs and 556 TEs were also observed in the candidate region associated with flower coloration. One pre-miRNA, miR118, affects flower pigment accumulation through ATP sulfurylase (Glazinska et al., 2019; Supplementary Tables S5–S7).

Other than color, the shapes of lotus flowers are also diverse. However, only one candidate region was found by GWAS (Supplementary Figure S3F; Supplementary Table S4). In this candidate region, the gene *Nn5g27350* with an annotated function of RNA binding was found, which also exhibited the highest expression in apical buds (Supplementary Figures S3F, S5C; Supplementary Table S4).

In our study, pairwise Pearson correlations displayed relatively high correlation coefficients (*r*) among the three traits, flower diameter, petal length and width (Figure 2A), which was consistent with the development of typical flowers, such as *Petunia* (Cao et al., 2018). 36, 17 and 19 predicted genes with known functions were significantly associated with flower diameter, petal length and petal width, respectively, which were near 38 peak SNPs in candidate regions. Nine of these genes had detailed known functions associated with flower-related traits, such as *GRF2* (*Nn3g18854*) with negative regulation of cell population proliferation and *NST1 (Nn3g18857)* with functions in plant-type cell wall biogenesis (Supplementary Figures S3G–I; Supplementary Table S4; Mitsuda et al., 2005; Liu et al., 2012). Moreover, five genes, *CBSX3* (*Nn1g09382*), *LKR* (*Nn8g38508*), *CLC-B* (*Nn4g22356*), *ATL57* (*Nn2g13886*), and *ZIGA4* (*Nn2g14730*) were primarily expressed in flower apical buds or petals. These proteins have crucial functions in cell proliferation and apoptosis, lysine synthesis, intracellular vacuole structure, protein ubiquitination and plastid development, which may influence cell number and growth (Supplementary Figures S5D–F; Supplementary Table S4; Zhu and Galili, 2003; Koussevitzky et al., 2007; Jenny et al., 2010; Shin et al., 2020). Further investigation of the relative effects of these candidate genes on cell number or cell size on petal morphogenesis is needed.

### Candidate genes associated with plant size variations

Plant size (architecture) is another crucial ornamental characteristic that is positively correlated with petal length, petal width and flower diameter (Figure 2B); plant size is determined by the coordinated progression of distinct organs based on the rate and duration of cell proliferation (Vercruyssen et al., 2011). A total of 54 genes located in 14 LD blocks that have known regulatory functions in the synthesis and transport of some proteins, cellular structure and jasmonate synthesis might be involved in the control of plant size in the development of lotus (Supplementary Figure S3J; Supplementary Table S4). *CLE26* (*Nn4g22977*), *ALA3* (*Nn4g22982*) and *VEL1* (*Nn8g40603*) were significantly associated with plant size variations involved in the regulation of cell differentiation in the phloem of roots and stems during auxin polar transport, vesicle-mediated transportation and the transition of the meristem from vegetative to reproductive phases (Supplementary Table S4; Kim and Sung, 2010; Czyzewicz and De Smet, 2016; Zhang X. et al., 2020). The *CLE26*, *ALA3* and *VEL1* genes were expressed primarily in the root apical meristem, immature stamen and root, respectively (Supplementary Figure S5G). This may be due to a close relationship between root growth and plant size. Notably, *ALA3* was likely a pleiotropic gene because it also had a significant effect on all three traits, petal length, petal width and flower diameter (Supplementary Table S3). *PRLIP* (*Nn3g19267*), belonging to the *PRLIP* family, which functions in the salicylic acid and ethylene signal transduction pathways, was expressed at the highest level in petioles (Jakab et al., 2003). Since petiole growth determines plant height, the petiole-expressed genes identified by GWAS might also be crucial in regulating plant size.

### Inference of the functions of trait-related genes by coexpression network analysis

**Figure 5:**
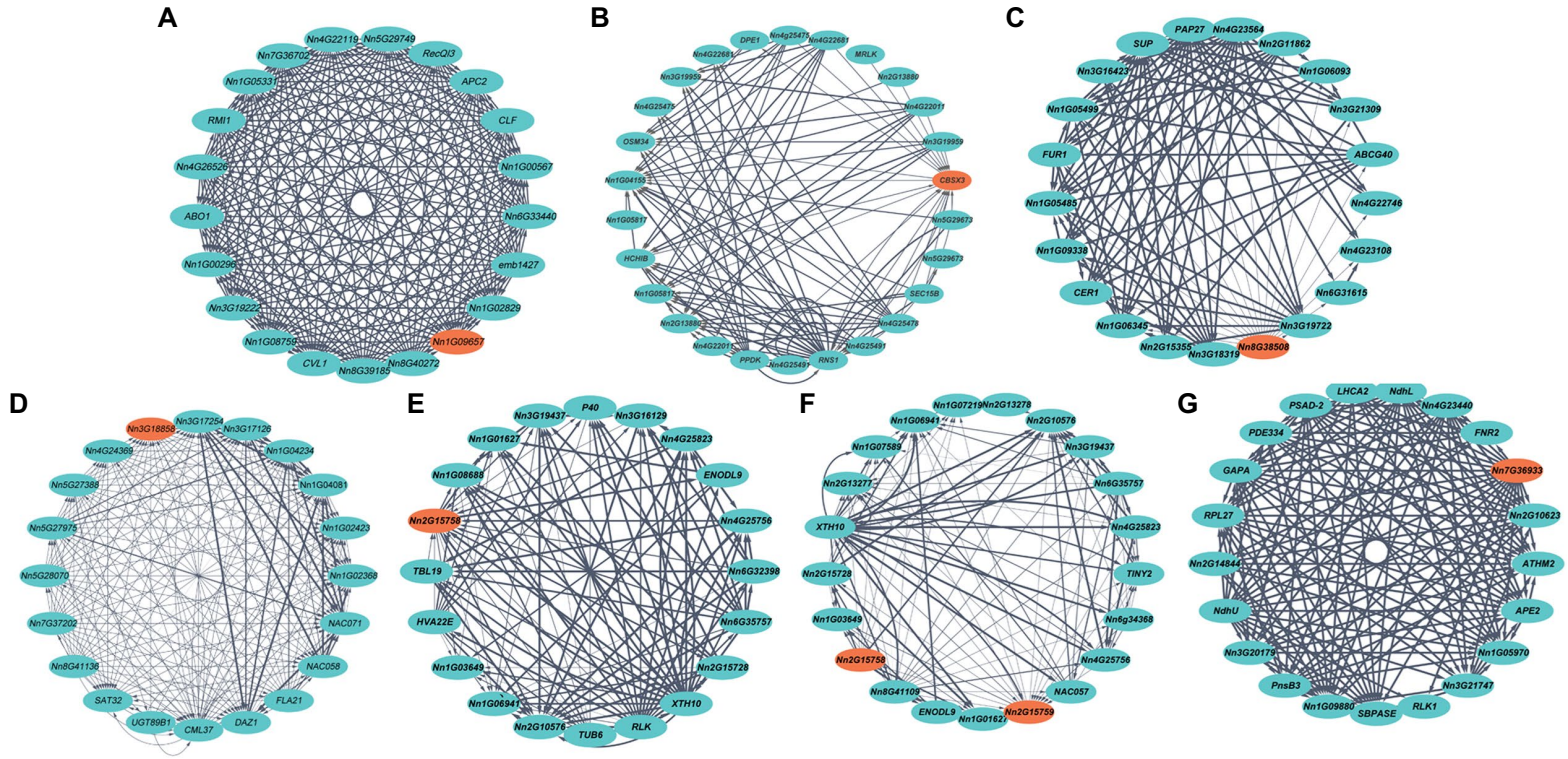
Inference of candidate gene functions by coexpression network analysis. Orange nodes denote trait-related candidate genes in our study. **(A-C)** Genes associated with flower diameter. **(D)** Genes associated with the maximum petal length. **(E,F)** Genes associated with the maximum petal width (cm). **(G)** Genes associated with plant size. Blue nodes are genes that are coexpressed with the candidate genes. The edge width denotes the interaction intensity between genes.

Gene coexpression networks based on diverse lotus tissues were downloaded from the *Nelumbo Genome Database* to identify genes coexpressed with trait-related genes from our GWAS (Figure 5). Seven genes associated with flower diameter, maximum petal length or width, and plant size were found in the GCNs, suggesting that gene variants associated with plant growth were highly coexpressed as networks in lotus. Among them, although the *Nn1g09657* did not have a known homolog, it was annotated with a role in the mitochondrial organization. It is coexpressed with *ABO1* (*Nn2g12928*; Nelissen et al., 2010), *RMI1* (*Nn4g22247*; Knoll and Puchta, 2011), and *APC2* (*Nn4g23232*; Lorenzo-Orts et al., 2019), which are involved in cell division, differentiation and proliferation and likely regulates flower diameter (Figure 5A). The second petal diameter-associated candidate gene, *CBSX3* (*Nn1g09382*), is coexpressed with *SEC15B* (*Nn5g29674*; Fendrych et al., 2010), *MRLK* (*Nn5g29797*), *DPE1* (*Nn1g05190*; Yazdanbakhsh and Fisahn, 2011), and *HCHIB* (*Nn3g19481*; Vahabi et al., 2013), which have roles in positively regulating the growth of pollen tubes and meristematic cells and sugar catabolic processes (Figure 5B). One petal diameter and plant size candidate gene, *LKR* (*Nn8g38508*), is coexpressed with genes such as *Nn1g05485* and *ABCG40* (*Nn2g11872*; Kang et al., 2015), which have roles in the ethylene biosynthetic process, jasmonic acid biosynthetic process and ABA transport (Figure 5C) and likely contribute to plant hormones and growth. One maximum petal length candidate gene, *Nn3g18858,* is coexpressed with other genes involved in calcium channel and cytoskeleton formation, such as *CML37* (*Nn1g09382*; Scholz et al., 2014; Figure 5D). Two maximum petal width-related genes, *Nn2g15758* and *Nn2g15759,* are coexpressed with *Nn4g25756*, *TUB6* (*Nn4g24212*; Soga et al., 2018), *Nn6g35757*, and *XTH10* (*Nn6g34564*; Sasidharan et al., 2010) and function in cell wall formation (Figures 5E,F). One plant size-related gene, *MPH1* (*Nn7g36933*), was associated with 20 genes (such as *LHCA2*, *RPL27*, *RLK1*) in a network controlling the synthesis of chloroplast proteins; because chloroplasts are present in most of the skin cells, this result is in line with the fact that *MPH1* had the greatest expression in the leaves (Figure 5G; Liu and Last, 2015).

## Discussion

### GWAS of flower lotus

Finding the genetic basis of complex traits in plants, such as organ morphogenesis, has been a particular focus in the study of the evolution and domestication of flowering plants. In addition to coding regions, genetic variants in non-coding regions, have also attracted increasing attention (Chen, 2009; Axtell, 2013). As an important flower in water gardening that has been grown for thousands of years in East Asia, lotus exhibits a plethora of trait diversity, particularly flowers. In this study of a cultivated lotus population with morphological diversity, we not only identified a series of candidate genes significantly associated with various traits in coding regions but also highlighted the importance of small RNAs and TEs in non-coding regions based on GWAS, particularly for the petaloid and petal number traits.

GWAS has successfully identified a polymorphism in the gene *DWARF8* that is responsible for earlier flowering in maize (Thornsberry et al., 2001). Its effectiveness in identifying candidate genes for 107 phenotypes in *Arabidopsis* has also been demonstrated (Atwell et al., 2010). More studies using high-throughput sequencing techniques and computational models to investigate plants with complex traits with greatly accelerate direct discoveries for causal variants in GWAS according to genes located within LD blocks (Brachi et al., 2011; Gupta et al., 2019; Alseekh et al., 2021). The high-quality and representative genome assembly of well-studied species can also help us identify SNP loci and candidate genes for GWAS (Ji et al., 2015). In this study, the marker density of lotus at the genome level, with an average of one SNP per 0.56 kb, was already high enough to complete GWAS compared to the 0.5 SNPs/kb in 107 *Arabidopsis* accessions (Atwell et al., 2010), 1.2 SNPs/kb for 224 flax accessions (Xie et al., 2018) and 1.3 SNPs/kb for 419 cotton accessions (Ma et al., 2018). A prerequisite of GWAS is the comprehensive characterization of the LD pattern depending on the standing genetic diversity and the number of recombination events in the populations (Brachi et al., 2011). In *Arabidopsis*, the optimal number of SNPs was estimated to be 140,000 when LD was shown to decay within 10 kb (Kim et al., 2007). Correspondingly, the level of LD decayed within 32.7 kb, 455,292 SNPs were found in our study, which was almost consistent with the predicted necessary number of SNPs. At the methodological level, different SL-GWAS models, like *GLM*, *MLM* and *CMLM*, can also affect the effectiveness of GWAS. In the present study, significant associations between SNP loci and phenotypic characteristics were detected in *GLM* (Q) but not found in *MLM* or *CMLM* (Q + K), as we demonstrated that one single population (*K* = 1) is optimal for our accessions. The core difference between *GLM*, *MLM* and *CMLM* is whether to control the kinship matrix *K*, and many studies have found that controlling *K* may lead to severe false negatives (Tibbs et al., 2021). To confirm the reliability of SNP loci in our study, the multi-locus GWAS (ML-GWAS) method with high detection power also needs to be considered, such as the study on the petal size of rapeseed (Qian et al., 2021). Generally, the number of significant SNPs associated with phenotypes by ML-GWAS was comparatively higher than those in the SL-GWAS model. A similar phenomenon could be found in the seed morphology of Brassica (280 SNP loci with ML-GWAS methods and 31 SNP loci with SL-GWAS methods; Khan et al., 2019) and plant architecture of hexaploid wheat (174 SNP loci with ML-GWAS methods, 97 SNP loci with SL-GWAS methods; Muhammad et al., 2021). This is likely because in ML-GWAS the effects of different loci in the whole genome can correct each other and then improve the detection efficiency of micro-effect loci. Also, this model generally does not form a series of significant SNPs, which will eliminate the collinearity caused by LD. Based on a suitable population, high marker density, and appropriate statistical models, GWAS has successfully explained the relationships between SNP markers and traits in other plants, such as inflorescence type in 82 *Hydrangea macrophylla* accessions (Wu and Alexander, 2020) and petal colors in 96 rose accessions (Schulz et al., 2016). In our study, we found a total of 149 possible candidate genes responsible for 12 traits, including flower development and plant size. There were also multiple high-quality SNPs in non-coding regions. Although only a few GWASs in plants have focused on non-coding regions with functional consequences associated with variants affecting the expression of nearby genes, abundant variations in non-coding regions can accelerate the evolution of traits with weaker selective constraints (Giral et al., 2018; Broekema et al., 2020). In maize, three microRNAs have shown a significant association with leaf architecture (Tian et al., 2011). Therefore, one future focus on GWAS in lotus populations will be to verify the functions of key elements in non-coding regions that are associated with gene regulation through systematic transcriptomic and functional-genomic studies.

Unlike other traits in our study, no SNP locus or candidate genes were found to have significant correlations with flowering time, population florescence, flower density and mean petal number per flower for lotus. Therefore, more populations and phenotypic data on flowering time and density should be included to study these traits.

### Molecular mechanisms underlying stamen petaloid formation

The normal formation of floral organs in each whorl of plants is directed by the classic ABCE model containing several *MADS*-box genes, which was discovered in *Arabidopsis* (Litt and Kramer, 2010). The aberrant development of the stamen in the second whorl leads to different flower types and has caused the evolution of lotus flower diversity, such as stamen petaloid. In previous studies, 11 homeotic *MADS*-box genes, one *AP2* gene and 31 methylation region-associated genes involved in stamen petaloid in lotus have been identified (Lin et al., 2018, 2019). Moreover, miR172, as a negative regulator, was also important during the crucial early stages of stamen development in lotus (Zhang Y. et al., 2020). However, the 16 candidate genes responsible for stamen petaloid variations in our study do not belong to the *MADS-box* family. They might be regulated in the downstream process by our GWAS-identified mutated genes responsible for flower type variations. Further molecular genetic studies are needed to investigate the regulatory relationship between our candidate genes and flower-type related *MADS-box* family.

Notably, we found novel structural variants in non-coding regions flanking these candidate genes responsible for stamen petaloid. The stamen petaloid in double-petalled flowers could be caused by a deleted fragment in the non-coding region that includes seven TEs (three MITEs) and two small RNAs (sRNAs). TEs are classified into retrotransposons (class I) and DNA transposons (class II) based on their transposition mechanisms (Wicker et al., 2007). Among most experimental populations, the changes in the numbers of TEs, as the most variable parts of the genome, may lead to phenotypic differences even among closely related plant species because these elements can move to different locations (Lisch, 2013). It is widely recognized that TEs, particularly MITEs, play a key role in the evolution of phenotypic diversity from seed pigment to flowering time in maize (Selinger and Chandler, 1999; Salvi et al., 2007). In the Japanese morning glory, MITEs could insert into some flavonoid-related structural genes (*CHS*, *DFR* and *F3’5’H*) and transcription factors (*bHLH*), resulting in the loss of pigment accumulation in flowers (Clegg and Durbin, 2000; Park et al., 2007), and an En/Spm-related TE inserted into the floral homeotic gene DP caused a double-flower phenotype (Nitasaka, 2003). MITEs have been suggested to be targets of small interfering RNAs (siRNAs) in rice, as a lower expression level was found in genes with MITE insertions (Lu et al., 2012). In our study, the gene expression level of *Nn5g26878* with MITE insertions in the promoter region was not detected in the few-petalled flower, which was in line with the assumption that the MITE-associated gene silencing resulted in normal stamen development in lotus. Generally, most sRNAs perfectly matching MITEs belonged to the 21 nt class, which has been shown in wheat (Cantu et al., 2010). However, 19 nt- and 23 nt-sRNAs in this non-coding region were found. In addition to MITEs, there are five other TEs without detailed information in our study. Mature sRNAs, especially siRNAs as defense machinery, can silence the expression of transposable elements in the genome through RNA–directed DNA methylation, which has been reported in multiple organisms, including wheat (Cantu et al., 2010), rice (Nosaka et al., 2012) and *Drosophila* (Czech et al., 2008). Given that siRNA-binding MITEs could induce methylation levels in nearby regions, the deletion of siRNA-associated MITEs in the region near *PRF* might reduce methylation and consequently induce gene expression linked to the stamen petaloid phenotype. This variant has also been confirmed by our reanalysis of another independent lotus population dataset and can be widely applied in future genetic and breeding projects.

## Data availability statement

These datasets presented in this study are deposited in the NCBI website (https://www.ncbi.nlm.nih.gov/). The accession numbers are SRP173547, SRP145546, SRP090666, PRJNA503979, PRJNA664744, and PRJNA417869.

## Author contributions

TS, XY, and JC conceived the project and designed the experiments. ZG, YL, and YW collected samples for whole-genome resequencing. ZG performed the methodology and the formal analysis and wrote the original draft. TS and YX revised and edited the manuscript. All authors contributed to the article and approved the submitted version.

## Funding

This work was supported by grants from the Strategic Priority Research Program of Chinese Academy of Sciences (No. XDB31000000), the Biological Resources Program, CAS (No. KFJ-BRP-007-009), the National Natural Science Foundation of China (Nos, 32170240, 31570220, and 31870208), the Youth Innovation Promotion Association of Chinese Academy of Sciences (No. 2019335), and Bureau of Landscaping and Forestry of Wuhan Municipality (No. WHGF2019A10).

## Conflict of interest

The authors declare that the research was conducted in the absence of any commercial or financial relationships that could be construed as a potential conflict of interest.

## Publisher’s note

All claims expressed in this article are solely those of the authors and do not necessarily represent those of their affiliated organizations, or those of the publisher, the editors and the reviewers. Any product that may be evaluated in this article, or claim that may be made by its manufacturer, is not guaranteed or endorsed by the publisher.

## Footnotes

^1^ http://nelumbo.biocloud.net

^2^ https://github.com/samtools/samtools

^3^ https://github.com/broadinstitute/picard

^4^ https://www.broadinstitute.org/gatk/

^5^ http://snpeff.sourceforge.net/

^6^ https://tassel.bitbucket.io/

^7^ https://www.arabidopsis.org/
