# Supplementary material for: Genome-wide association study of traits in sacred lotus uncovers MITE-associated variants underlying stamen petaloid and petal number variations
Source: Front Plant Sci. 2022 Sep 23;13:973347. doi: 10.3389/fpls.2022.973347 (PMC9539442; doi:10.3389/fpls.2022.973347)
Supplement: Supplementary file 1 [file Data_Sheet_1.PDF]

Genome-wide association study of traits in sacred lotus uncovers MITE-associated variants underlying stamen petaloid and petal number variations

Running title: GWAS of flower lotus

Zhiyan Gao[1,2,3], Yuting Liang[4], Yuhan Wang[5], Yingjie Xiao[6], Jinming Chen[1,2], Xingyu Yang[4*], Tao Shi[1,2*]

[1] Key Laboratory of Aquatic Botany and Watershed Ecology, Wuhan Botanical Garden, Chinese Academy of Sciences, Wuhan 430074, China

[2] Center of Conservation Biology, Core Botanical Gardens, Chinese Academy of Sciences, Wuhan 430074, China

[3] University of Chinese Academy of Sciences, Beijing 100049, China

[4] Wuhan Institute of Landscape Architecture, Wuhan 430081, China

[5] Wuhan Institute of Design and Sciences, Wuhan 430205, China

[6] National Key Laboratory of Crop Genetic Improvement, Huazhong Agricultural University, Wuhan, 430070, China

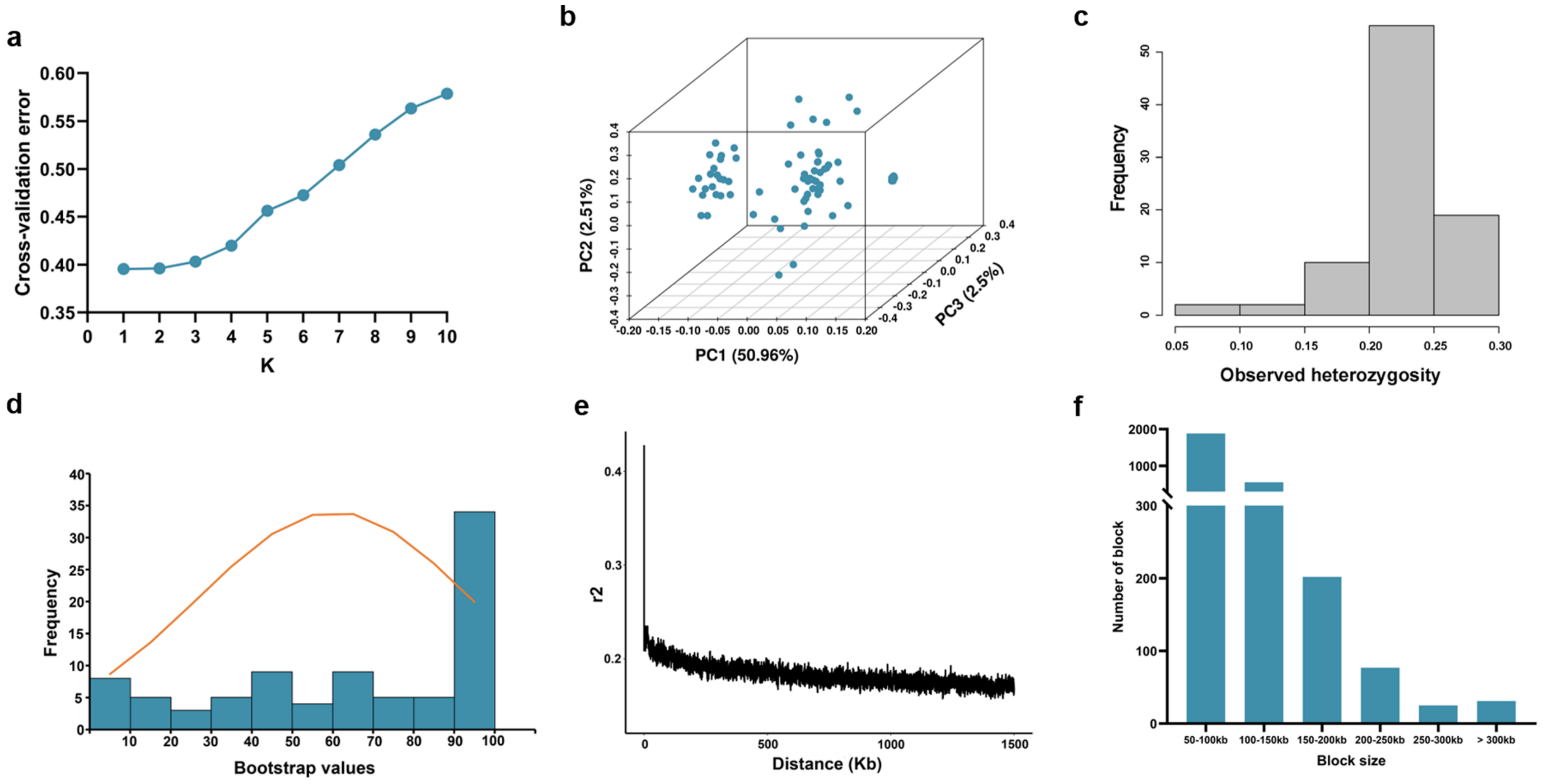

Fig. S1 Genetic structure, PCA and LD decay for the high-quality SNPs in the 88 cultivated lotus accessions. (a) Structure analysis from K = 1 to K=10. (b) 3D PCA plot of the first, second and third components (PC1, PC2 and PC3). (c) Frequency distribution of observed heterozygosity in 88 lotus accessions. (d) The histogram shows the distribution of bootstrap values in the phylogenetic tree. (e) Genome-wide average LD decay estimated from the whole population. (f) Distribution of LD block size in the whole genome.

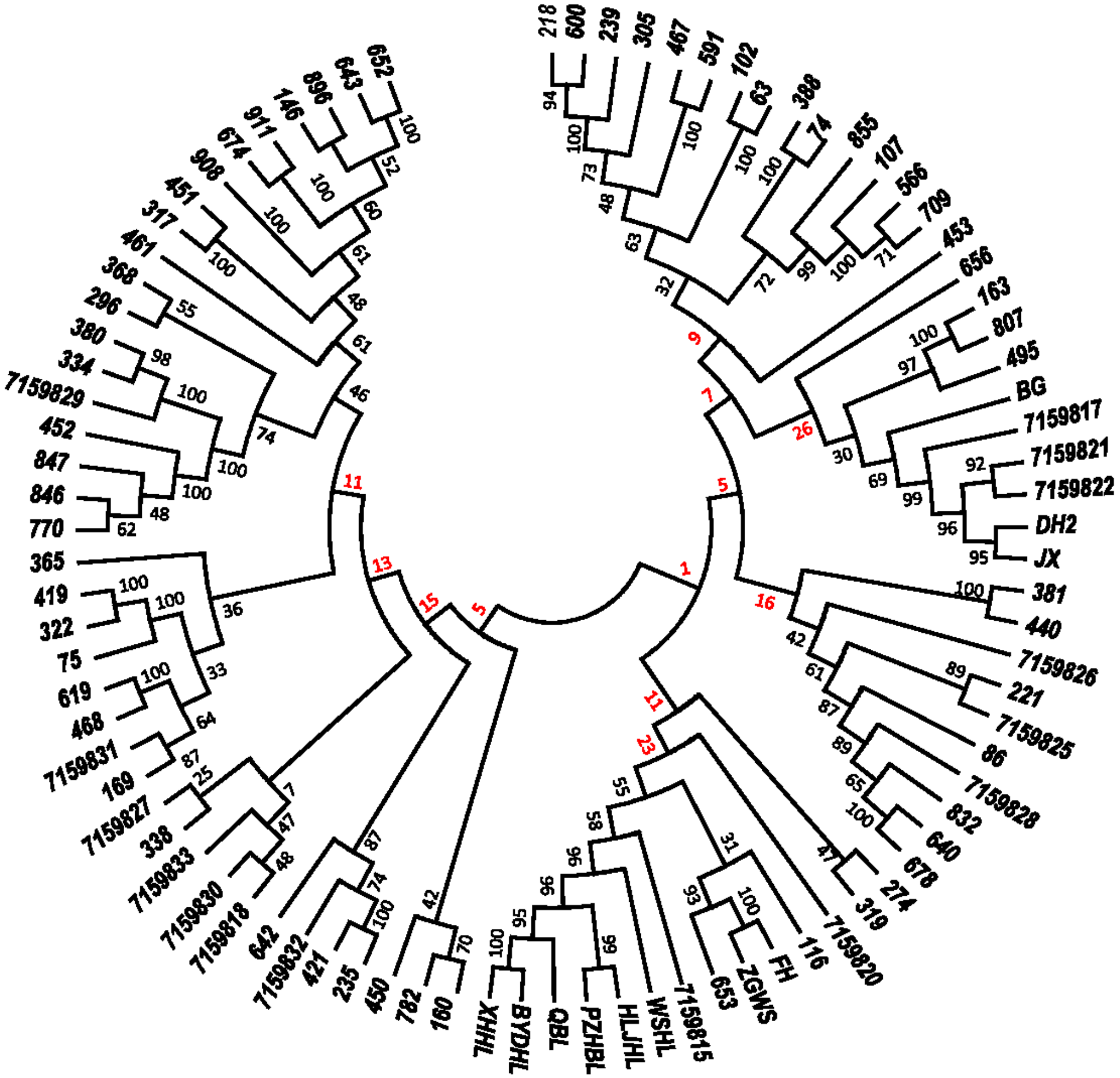

Fig. S2 Phylogenetic tree showing the phylogenetic relationships among the 88 lotus accessions in this population. Red numbers represent low bootstrap values in early-branching nodes.

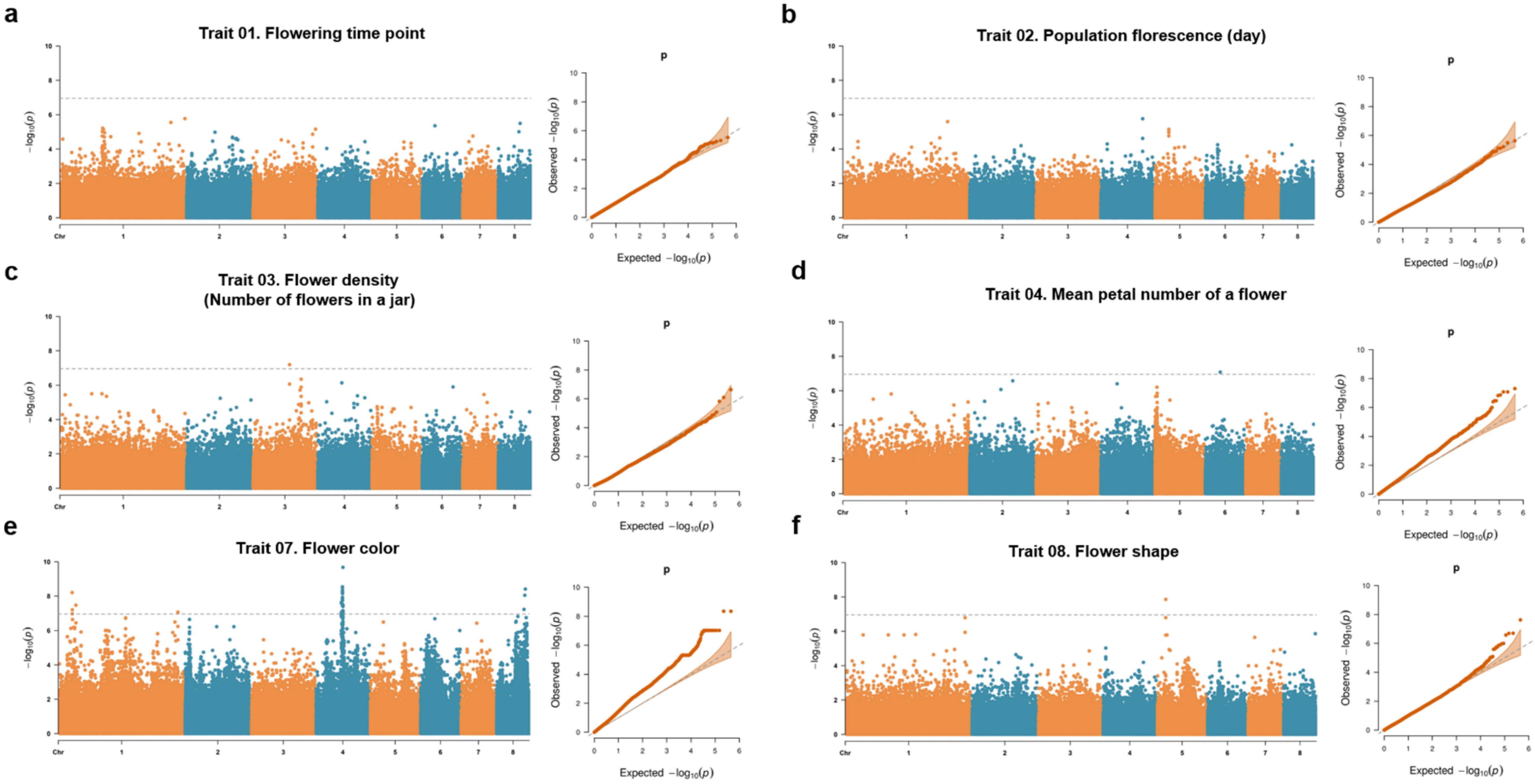
a
Trait 01. Flowering time point
b
Trait 02. Population florescence (day)
c
Trait 03. Flower density
(Number of flowers in a jar)
d
Trait 04. Mean petal number of a flower
e
Trait 07. Flower color
f
Trait 08. Flower shape
Chr
$-\log_{10}(p)$
p
Observed $-\log_{10}(p)$
Expected $-\log_{10}(p)$

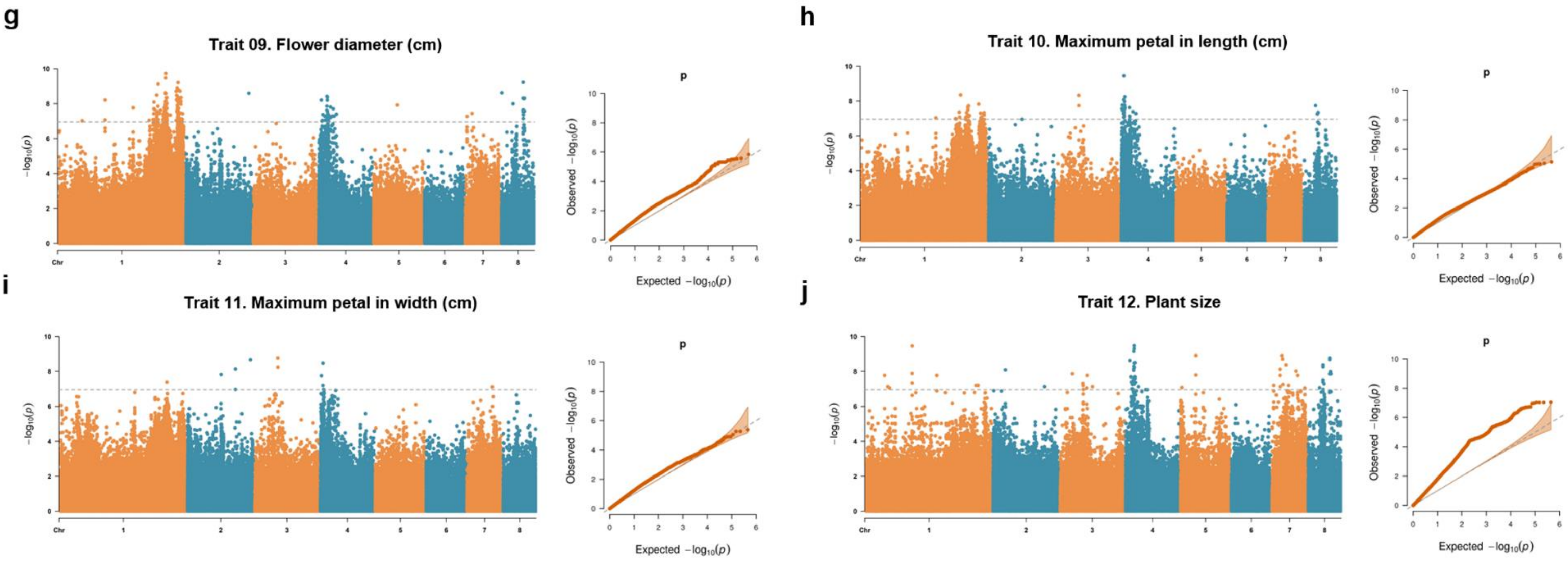

Fig. S3 Manhattan plots and quantile-quantile plots for the genome-wide association study (GWAS) of 10 traits in 88 lotus accessions using a generalized linear model (*GLM*). The gray dotted line represents the significance level (*, -log10 *P* = 6.9, *P* value < 1.106378e-07).

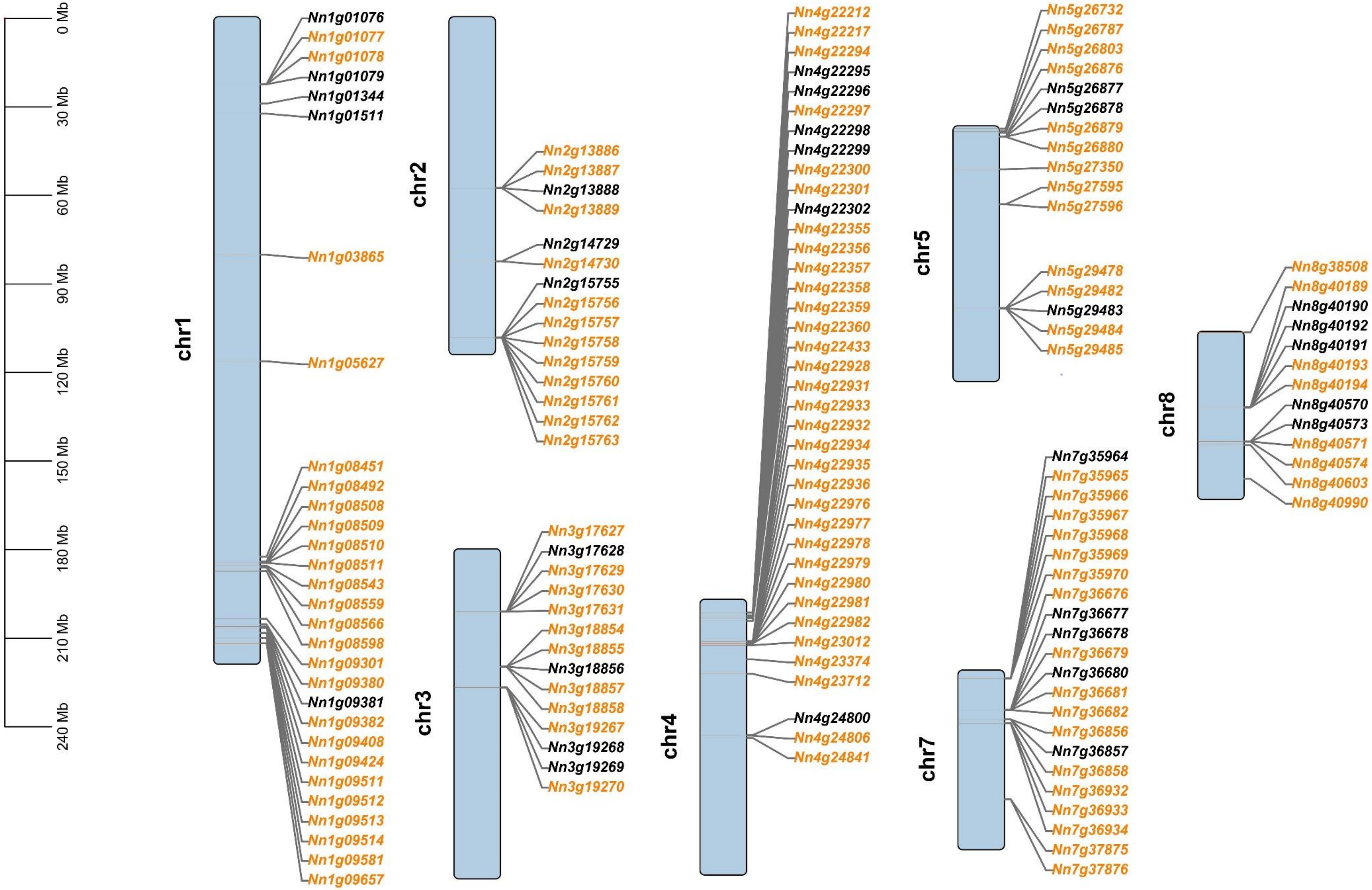

Fig. S4 The distribution of significant trait-related genes on eight chromosomes. The genes in yellow represent genes with known functions, and those in black represent genes with unknown functions.

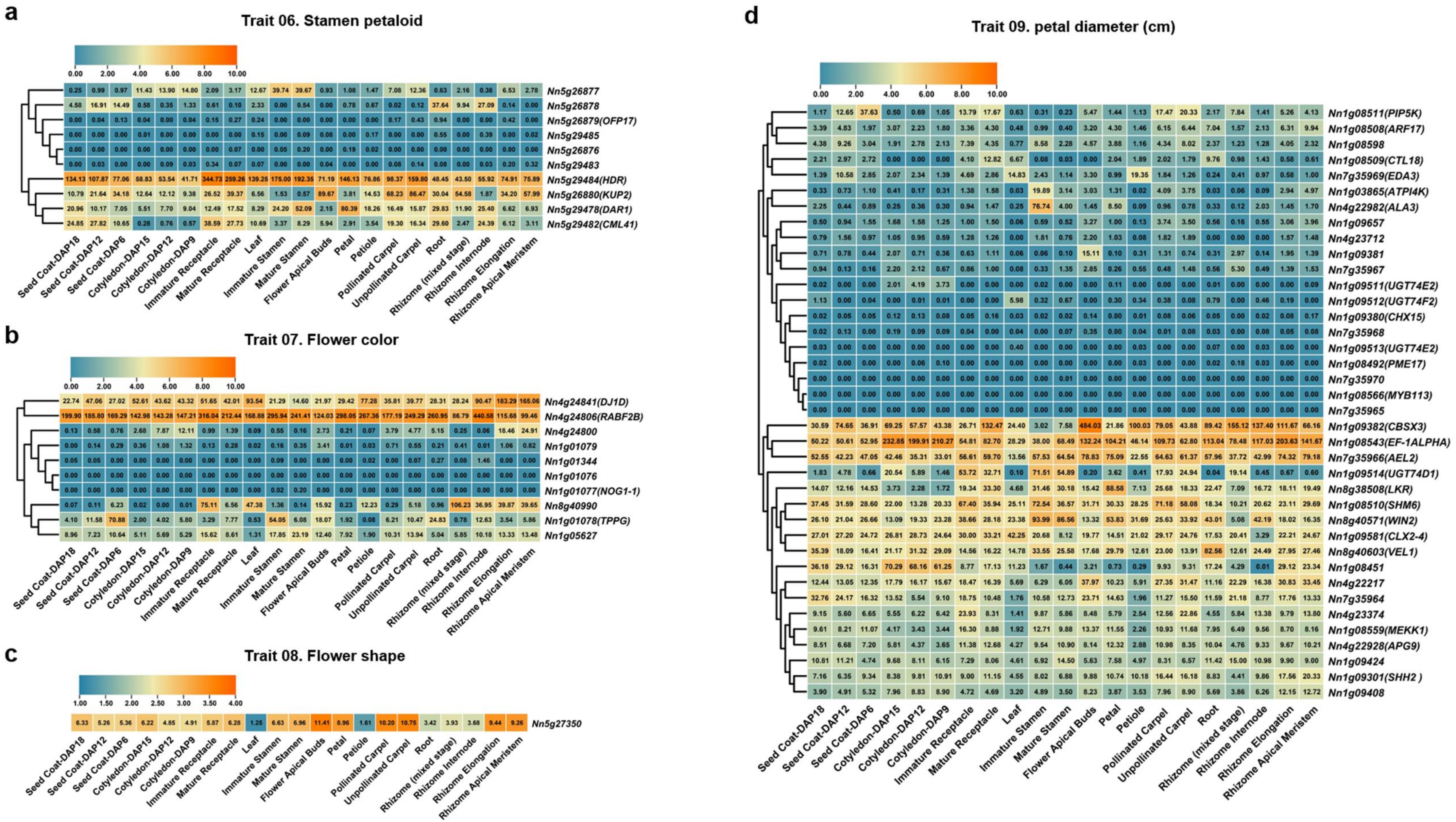
a
Trait 06. Stamen petaloid
0.00 2.00 4.00 6.00 8.00 10.00
Nn5g26877
Nn5g26878
Nn5g26879(OFP17)
Nn5g29485
Nn5g26876
Nn5g29483
Nn5g29484(HDR)
Nn5g26880(KUP2)
Nn5g29478(DAR1)
Nn5g29482(CML41)
b
Trait 07. Flower color
Nn4g24841(DJ1D)
Nn4g24806(RABF2B)
Nn4g24800
Nn1g01079
Nn1g01344
Nn1g01076
Nn1g01077(NOG1-1)
Nn8g40990
Nn1g01078(TPPG)
Nn1g05627
c
Trait 08. Flower shape
1.00 1.50 2.00 2.50 3.00 3.50 4.00
Nn5g27350
d
Trait 09. petal diameter (cm)
Nn1g08511(PIP5K)
Nn1g08508(ARF17)
Nn1g08598
Nn1g08509(CTL18)
Nn7g35969(EDA3)
Nn1g03865(ATPI4K)
Nn4g22982(ALA3)
Nn1g09657
Nn4g23712
Nn1g09381
Nn7g35967
Nn1g09511(UGT74E2)
Nn1g09512(UGT74F2)
Nn1g09380(CHX15)
Nn7g35968
Nn1g09513(UGT74E2)
Nn1g08492(PME17)
Nn7g35970
Nn1g08566(MYB113)
Nn7g35965
Nn1g09382(CBSX3)
Nn1g08543(EF-1ALPHA)
Nn7g35966(AEL2)
Nn1g09514(UGT74D1)
Nn8g38508(LKR)
Nn1g08510(SHM6)
Nn8g40571(WIN2)
Nn1g09581(CLX2-4)
Nn8g40603(VEL1)
Nn1g08451
Nn4g22217
Nn7g35964
Nn4g23374
Nn1g08559(MEKK1)
Nn4g22928(APG9)
Nn1g09424
Nn1g09301(SHH2)
Nn1g09408
Seed Coat-DAP18
Seed Coat-DAP12
Seed Coat-DAP6
Cotyledon-DAP15
Cotyledon-DAP12
Cotyledon-DAP9
Immature Receptacle
Mature Receptacle
Leaf
Immature Stamen
Mature Stamen
Flower Apical Buds
Petal
Petiole
Pollinated Carpel
Unpollinated Carpel
Root
Rhizome (mixed stage)
Rhizome Internode
Rhizome Elongation
Rhizome Apical Meristem

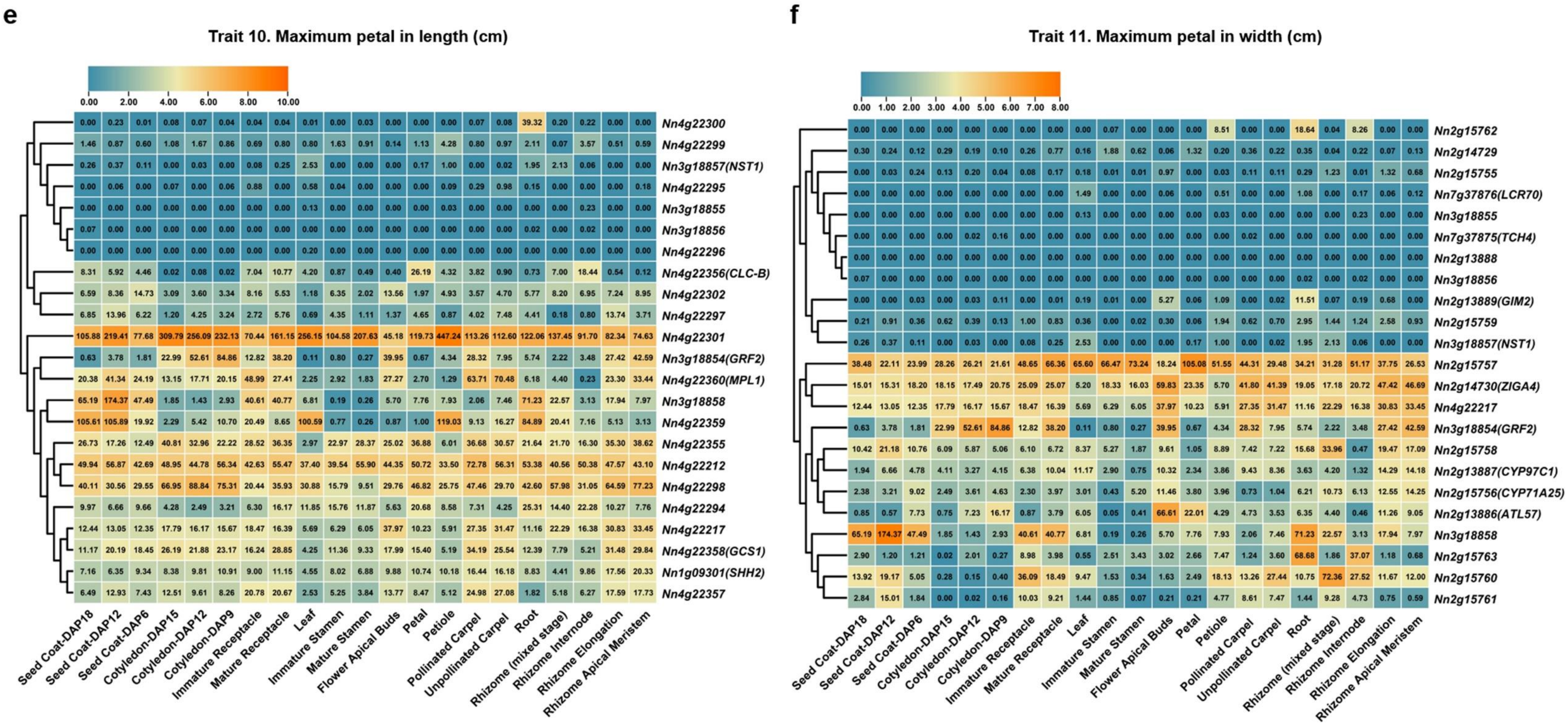

e
Trait 10. Maximum petal in length (cm)
0.00 2.00 4.00 6.00 8.00 10.00
Nn4g22300
Nn4g22299
Nn3g18857(NST1)
Nn4g22295
Nn3g18855
Nn3g18856
Nn4g22296
Nn4g22356(CLC-B)
Nn4g22302
Nn4g22297
Nn4g22301
Nn3g18854(GRF2)
Nn4g22360(MPL1)
Nn3g18858
Nn4g22359
Nn4g22355
Nn4g22212
Nn4g22298
Nn4g22294
Nn4g22217
Nn4g22358(GCS1)
Nn1g09301(SHH2)
Nn4g22357
Seed Coat-DAP18
Seed Coat-DAP12
Seed Coat-DAP6
Cotyledon-DAP15
Cotyledon-DAP12
Cotyledon-DAP9
Immature Receptacle
Mature Receptacle
Leaf
Immature Stamen
Mature Stamen
Flower Apical Buds
Petal
Petiole
Pollinated Carpel
Unpollinated Carpel
Root
Rhizome (mixed stage)
Rhizome Internode
Rhizome Elongation
Rhizome Apical Meristem
f
Trait 11. Maximum petal in width (cm)
0.00 1.00 2.00 3.00 4.00 5.00 6.00 7.00 8.00
Nn2g15762
Nn2g14729
Nn2g15755
Nn7g37876(LCR70)
Nn3g18855
Nn7g37875(TCH4)
Nn2g13888
Nn3g18856
Nn2g13889(GIM2)
Nn2g15759
Nn3g18857(NST1)
Nn2g15757
Nn2g14730(ZIGA4)
Nn4g22217
Nn3g18854(GRF2)
Nn2g15758
Nn2g13887(CYP97C1)
Nn2g15756(CYP71A25)
Nn2g13886(ATL57)
Nn3g18858
Nn2g15763
Nn2g15760
Nn2g15761
Seed Coat-DAP18
Seed Coat-DAP12
Seed Coat-DAP6
Cotyledon-DAP15
Cotyledon-DAP12
Cotyledon-DAP9
Immature Receptacle
Mature Receptacle
Leaf
Immature Stamen
Mature Stamen
Flower Apical Buds
Petal
Petiole
Pollinated Carpel
Unpollinated Carpel
Root
Rhizome (mixed stage)
Rhizome Internode
Rhizome Elongation
Rhizome Apical Meristem

g

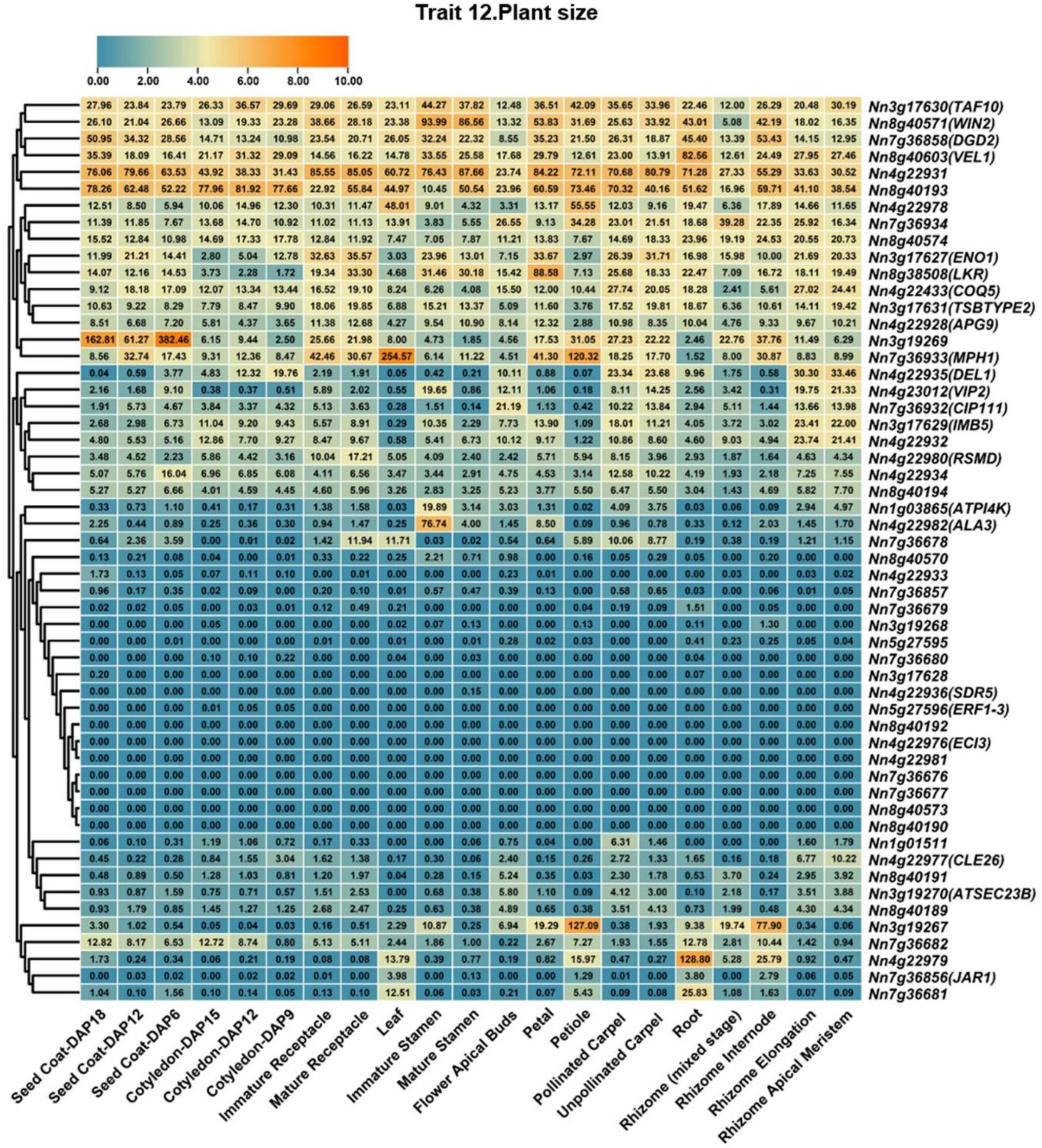

Fig. S5 Heatmap and hierarchical clustering showing the expression pattern of trait-related genes in different tissues. The expression values were scaled and centered in cells of heatmap. The orange to blue gradient indicates high expression to low expression.

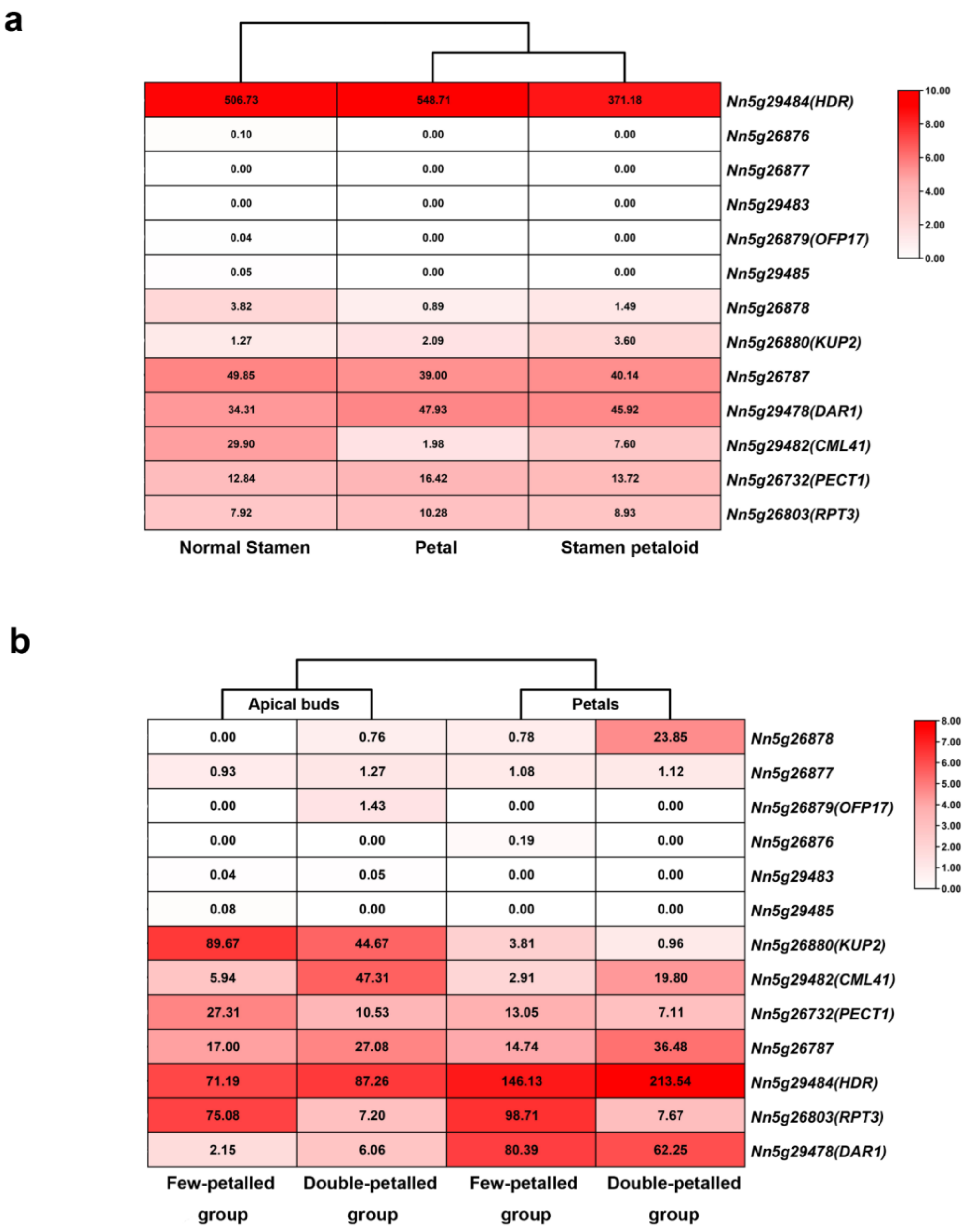

Fig. S6 Heatmap and clustering showing the expression pattern of trait-related genes for stamen petaloid and flower types. (a) The comparison of trait-related genes among petal, normal stamen and stamen petaloid tissues from the same double-petalled group (Lin et al., 2019). (b) The comparison of trait-related genes between few-petalled lotus group (Li et al., 2021) and double-petalled lotus group.

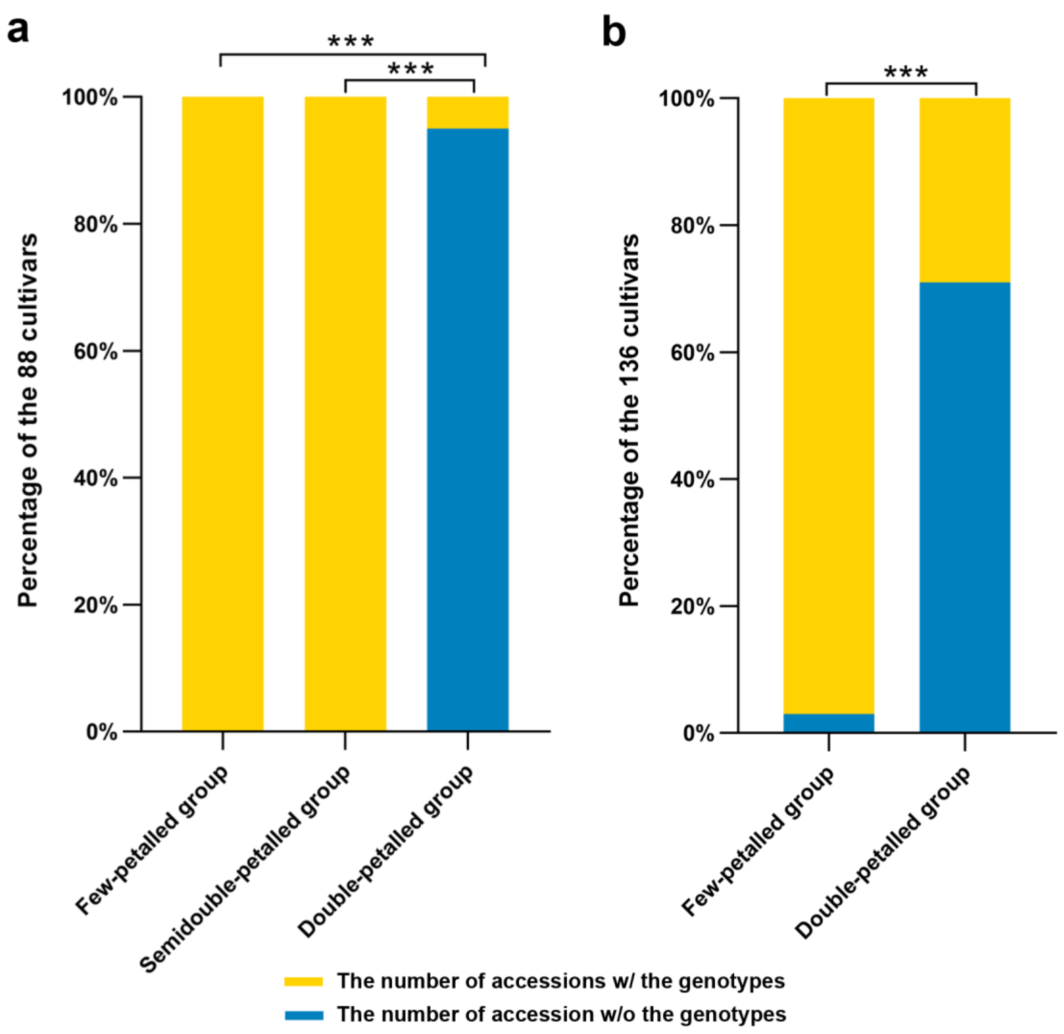

Fig.S7 The proportion of lotus accessions with genomic fragments that are missing and present for the region (3,670,710- 3.673,465 on chromosome 5) of 88 lotus individuals in our study (a) and 136 lotus accessions with high-quality resequencing datasets in another study (Liu et al., 2020) (b) based on delly analysis of deletion (***, *P* value < 0.001).
